# Evaluating a brief smartphone-based stress management intervention with heart rate biofeedback from built-in sensors in a three arm randomized controlled trial

**DOI:** 10.1038/s41598-025-06588-4

**Published:** 2025-06-23

**Authors:** Lukas M. Fuhrmann, Christian Aljoscha Lukas, Lena Schindler-Gmelch, Matthias Berking

**Affiliations:** 1 Department of Clinical Psychology and Psychotherapy, Friedrich-Alexander Universität Erlangen-Nürnberg, Nägelsbachstraße 25a, 91052 Erlangen, Germany; 2 mentalis GmbH, Nuremberg, Germany

**Keywords:** Stress management, Smartphone-based app brief intervention, Smartphone sensors, Biofeedback, Signal-processing, Psychology, Quality of life

## Abstract

Perceived stress is prevalent in industrial societies, negatively impacting mental health. Smartphone-based stress management interventions provide accessible alternatives to traditional methods, but their efficacy remains modest, potentially due to limited integration of smartphone sensor technology. The primary aim of this study was to evaluate the efficacy of an 18-day smartphone-based stress management intervention, *MT-StressLess* with integrated heart rate (HR)-based biofeedback using built-in accelerometer sensors, compared to a waitlist control (WLC) condition. Secondary outcomes included emotion regulation skills, depressive symptoms, overall well-being, usbiality and usage data. As exploratory aims, we investigated whether the *MT-StressLess* version without HR-based biofeedback was also superior to the WLC condition, and whether the version with HR-based biofeedback provided additional benefits compared to the version without. In a three-arm randomized controlled trial, 166 participants were assigned to *MT-StressLess* with HR-based biofeedback, *MT-StressLess*, or the WLC condition. Linear mixed-effects models were used to analyze intervention effects over time (baseline, postintervention, and 1-month follow-up). At postintervention, *MT-StressLess* with HR-based biofeedback showed significantly greater reductions in perceived stress compared to the WLC condition (*d* = 0.41, 95% CI [0.03, 0.79]), whereas the version without biofeedback did not differ significantly (*d* = 0.14, 95% CI [−0.24, 0.51]). No significant differences were observed between the two active conditions (*d* = 0.29, 95% CI [−0.08, 0.66]). Both active conditions, however, led to significant improvements in the secondary outcomes of emotion regulation skills and well-being compared to the WLC (all *ds* = −0.58 to −0.27). These patterns persisted at the 1-month follow-up. Usability ratings were high, but overall adherence was moderate. The findings in the main comparison may reflect increased interoceptive awareness and self-regulation. Yet, the limited effects of the core intervention and the biofeedback component also suggest the influence of non-specific factors, such as placebo effects, outcome expectancy and user engagement, which highlights the need to better understand optimal intervention duration, motivation, reinforcement, and more individualized approaches to stress reactivity. Overall, the findings provide preliminary support for the potential of a smartphone-based intervention that includes HR-based biofeedback to reduce perceived stress compared to no intervention. As these interventions are still in their early stages, future research should explore how personalization driven by artificial intelligence and real-time physiological tracking can enhance engagement and efficacy.

**Supplementary Information:**

The online version contains supplementary material available at 10.1038/s41598-025-06588-4.

## Introduction

The number of individuals affected by stress and stress-related problems continues to increase in industrialized societies, which are characterized by high demands at work and in daily life^1–3^. Research indicates that stress is associated with the development and maintenance of psychological disorders such as depression, anxiety, substance use, and sleep disorders, as well as physical health problems, such as cardiovascular diseases and obesity^4,5^. In addition to the individual burden, stress-related health problems cause high direct and indirect societal costs due to greater incidence of employee sick leave, staff turnover, reduced productivity, and increased health care costs^6,7^. Given the widespread impact of stress, there is an urgent need for effective, scalable, and accessible interventions to facilitate successful stress management.

Numerous models have been developed to examine the multifaceted nature of stress. The Transactional Model of Stress and Coping by Lazarus and Folkman (1984)^8^ conceptualizes stress as a dynamic interaction between an individual and their environment, highlighting the central role of cognitive appraisals in evaluating the significance of events and the resources available for coping. Based on this framework, stress management interventions typically aim either to modify individuals’ appraisals, for example, through cognitive reappraisal^9^, or to enhance their perceived ability to cope, using techniques such as muscle relaxation^10^, breathing exercises^11^, and interoceptive awareness, defined as the perception and interpretation of internal bodily signals^12,13^. Interoceptive awareness, in particular, plays a crucial role in enabling individuals to recognize physiological stress cues early and respond with effective coping strategies^14,15^. Through the adoption of such techniques, individuals may improve their emotion regulation capacities, thereby supporting long-term psychological well-being^16^.

With regard to existing treatments, several meta-analyses have demonstrated the efficacy of face-to-face psychological stress management interventions. For instance, such interventions have shown moderate-to-large effect sizes in reducing stress among employees (*g* = 0.25–0.77)^17^ and for college students (*g* = 0.58, 95% CI [0.44, 0.73])^18^. These interventions typically include structured programs aimed at enhancing coping mechanisms and building resilience. For employees, face-to-face stress management interventions often consist of workshops, elements of cognitive-behavioral therapy (CBT), mindfulness programs, and peer support groups, frequently integrated into workplace wellness initiatives. Similarly, university counseling services provide psychoeducational programs, individual or group counseling, and tailored workshops that address personal, academic, or career-related stressors^17,18^.

Despite the demonstrated efficacy of face-to-face stress management interventions, their widespread adoption is hindered by practical barriers, including restricted access to specialized care, fear of stigmatization, and difficulties in allocating sufficient time for participation^19,20^. Smartphone-based stress management interventions have emerged as promising alternatives that offer greater accessibility, anonymity, and convenience. These features help mitigate many of the challenges associated with traditional interventions, making smartphone-based approaches particularly appealing. However, their overall efficacy remains modest. A recent meta-analysis by Linardon and colleagues (2024) reported a small effect size (*g* = 0.29, 95% CI [0.16, 0.43]) across 33 randomized controlled trials (RCTs) evaluating smartphone-based interventions targeting stress symptoms^21^.

Most existing app-based interventions primarily rely on automated psychoeducation, structured guidance through stress-management exercises, symptom monitoring, and remote support from E-coaches, who provide tailored feedback to enhance motivation and facilitate the application of learned strategies^21,22^. Factors such as low adherence rates, lack of engagement, and passive content delivery are likely to contribute to the modest efficacy of app-based stress management interventions. Another key limitation may be the insufficient integration of smartphone technology, particularly built-in sensing and machine-learning techniques, into existing intervention frameworks^23^. By incorporating mobile automated assessments of physiological indicators, such as pupil dilation^24^, blood volume changes^25^, or breathing patterns^26^, these interventions could provide more precise, real-time feedback on users’ stress levels. Among physiological stress markers, heart rate (HR) is particularly relevant for smartphone-based biofeedback applications^27^, because it (a) reliably reflects autonomic nervous system activity^28^, (b) has been effectively utilized in desktop-based biofeedback interventions^29^, (c) has been validated as a stress indicator in mobile applications^30^, and (d) provides users with tangible signals to recognize stress, increase interoceptive awareness, and practice relaxation techniques such as deep breathing or guided imagery^15,29,31^. By integrating physiological data with coping strategies, HR biofeedback may help translate stress awareness into adaptive behavioral responses, offering a feasible approach for stress management.

Encouraging findings from adjacent research areas suggests that app-based interventions incorporating biofeedback can be beneficial. For instance, studies have used external optical measurement methods such as photoplethysmography sensors attached to the participants’ earlobe^32^, fingertips^32^, or wrists^33,34^. In a quasi-experimental study by Economides and colleagues^32^, the investigators demonstrated that a smartphone-based intervention combining daily HR variability (HRV) feedback with breathing exercises was more effective in reducing depressive symptoms than an active control condition in which participants were instructed to apply the same breathing techniques without receiving HRV feedback. In another (albeit uncontrolled) study, Latour and colleagues found a decrease in PTSD symptoms, depression, and alcohol use among military veterans who met the criteria for PTSD after participating in a smartphone-based intervention involving continuous HR feedback^33^. Finally, in an RCT, university students equipped with a wearable mobile biofeedback device that monitored their HR, sleep, and physical activity and presented these data to them via their smartphones reported a significantly greater reduction in anxiety and depression compared with students assigned to a waitlist control (WLC) condition^34^.

Despite these promising findings, the integration of external monitoring devices presents several challenges, including technical complexity, reduced adherence due to cumbersome setups, and increased costs^35^. Previous research indicates that adherence rates to wearable-based biofeedback interventions decline due to discomfort, maintenance burden, and low usability^36^. A more user-friendly alternative is to leverage built-in smartphone sensors. This advancement eliminates the need for external devices such as wristbands, electrodes, or additional hardware, thereby reducing setup time, minimizing movement restrictions, and avoiding potential discomfort or adverse skin reactions associated with conventional sensors^37,38^. By integrating HR biofeedback into smartphone interventions, users can receive immediate personalized feedback on their stress levels and coping strategies. This approach has considerable potential for enhancing user engagement and improving the overall efficacy of app-based stress-management strategies.

However, there is a lack of studies evaluating the efficacy of therapeutic applications that utilize built-in smartphone for HR-based biofeedback in reducing stress. Among the 33 RCTs reviewed by Linardon and colleagues, only two interventions incorporated built-in sensory features into their stress-management interventions, and only one focused on HRV. Yoon and colleagues measured HRV via photoplethysmography using a mobile camera before delivering mindfulness content, but no significant differences were found between the intervention and WLC condtion^39^. However, this lack of effect may be attributable to the small sample size (*n* = 45), which resulted in insufficient statistical power to detect meaningful effects. Furthermore, limitations in measurement accuracy are possible because smartphone-based photoplethysmography can be influenced by factors such as ambient light^40^, skin tone^41^, and motion artifacts^42^. One promising approach to overcome this limitation is to utilize smartphone accelerometers to extract HR indicators. These sensors measure acceleration forces across three axes (x, y, and z) and are traditionally used for motion detection and screen orientation, but they can also provide insights into physiological data^43^. To the best of our knowledge, no study to date has employed accelerometers for HR biofeedback in the context of stress.

This trial aimed to evaluate the efficacy of an 18-day smartphone-based stress management intervention based on CBT combined with accelerometer-derived HR biofeedback. Following extensive feasibility testing and consideration of usability and adherence information, we compared a stress-management app intervention based on CBT with HR-based biofeedback to a WLC condition. Our primary hypothesis was that participants using the app-based intervention with HR-based biofeedback would experience greater reductions in perceived stress than those in the WLC condition over time. Furthermore, we expected greater improvements in secondary outcomes, including emotion regulation skills, depressive symptoms, and overall well-being. In addition, given the limited research on stress-focused app-based intervention studies that systematically evaluatedg specific intervention components, we included an otherwise identical app-based intervention without HR-based biofeedback as an exploratory condition. This allowed us to examine whether this version would also lead to greater stress reduction and improvements in secondary outcomes compared to the WLC condition and to explore whether adding HR-based biofeedback would provide additional benefits over the intervention without biofeedback.

## Methods

### Study design

This study is reported in compliance with the CONSORT-EHEALTH statement^44^. To test our hypotheses, participants were randomly allocated to one of three study conditions (*Mentalis StressLess* app (*MT-Stressless*) with HR-based biofeedback vs. *MT-Stressless* without HR-based biofeedback vs. WLC) between August and December 2017. Prior to the study, extensive feasibility testing was conducted with a total of *n* = 17 healthy individuals. As a first step, in an HR-focused pre-assessment (*n* = 9, *M* age = 28.5 years, *SD* = 12.3, gender: *n* = 4 females), we confirmed that the assessment of HR using the smartphone accelerometer was feasible and sufficiently accurate as compared to conventional electrocardiography (see Supplementary Material, Figure S1 and Table S1) and determined adequate cutoff values for stress and relaxation phases in a relaxation exercise. As a second step, in an app-focused pre-assessment (*n* = 8, *M* age = 36.94 years, *SD* = 17.03, gender: *n* = 4 females), we evaluated software functionality properly and implemented targeted optimizations (see 2. Approach-Avoidance Modification Training). The study was conducted in accordance with the Declaration of Helsinki and approved by the Ethics Committee of the German Psychological Society (registration number: DGPS; MB 092017_amd_072016). The trial was retrospectively registered with the German Register for Clinical Trials under DRKS00013073 (registration date: 21/02/2018) shortly after data collection was completed and prior to the data analysis.

### Sample size and power

According to our a priori power analysis conducted with G*Power 3.1.9.7^45^ at least *n* = 53 participants per condition would be required to provide sufficient power to test a medium effect of *d* = 0.50 in an analysis of covariance (ANCOVA) for the primary outcome, with three conditions and one covariate at a significance level of 0.05 with a power of 0.80. Because there were no comparable studies with two active smartphone intervention conditions and a WLC condition, we assumed a medium effect size for the overall ANCOVA across all three conditions rather than focusing on the difference between the two active conditions. This assumption was based on effect sizes reported at the time the study was designed, as observed by Heber and colleagues^46^ (*d* = 0.43, 95% CI [0.31, 0.54])^46^ and Ly and colleagues (*d* = 0.50 (95%, CI [−1.29, 2.29])^47^.

To better match the data structure, final analyses were conducted using Linear Mixed Models (LMMs). Accordingly, we conducted a post hoc power analysis to determine the smallest effect size (f^2^) that could be detected with 80% power (1−β = 0.80) at α = 0.05 in our sample (*N* = 159). Given two fixed effects (Condition and Condition × Time), the degrees of freedom for the error variance were v = 156. The analysis revealed that the study was adequately powered to detect an effect size of f^2^ = 0.062, corresponding to Cohen’s d of approximately 0.21, and thus, a small effect.

### Participants and procedures

We recruited participants from the general population via social media, flyers, and bulletin board advertisements. Recruitment was based on the premise that individuals willing to participate in a stress intervention study would experience a meaningfully increased level of perceived stress. As an additional incentive for participation, participants were offered the chance to win one of four 25 € online store vouchers. Alternatively, they could gain course credits if they were studying psychology at the university hosting the study. Interested individuals were provided with a link or QR code that directed them to a website containing general study information. After agreeing to the terms and conditions for participating in the screening, they completed questionnaires online (via LimeSurvey). In the screening, the following inclusion criteria were assessed: (a) minimum age of 18 years, (b) access to a smartphone using Android 4.4 or newer, and (c) sufficient German language skills. In addition, the current perceived stress level, sociodemographic variables (e.g., gender, age, education, employment), health-related information (e.g., current psychotherapeutic or psychiatric treatment, current cardiac problems, average estimated weekly hours of physical activity), previous experience with relaxation procedures such as progressive muscle relaxation, autogenic training, yoga, meditation, as well as the daily estimated average amount of time dedicated to smartphone use were assessed. Eligible participants received detailed study information via email, along with a consent form to be signed and returned prior to participation. Written informed consent was obtained from all participants and/or their legal guardian(s).

**Fig. 1:**
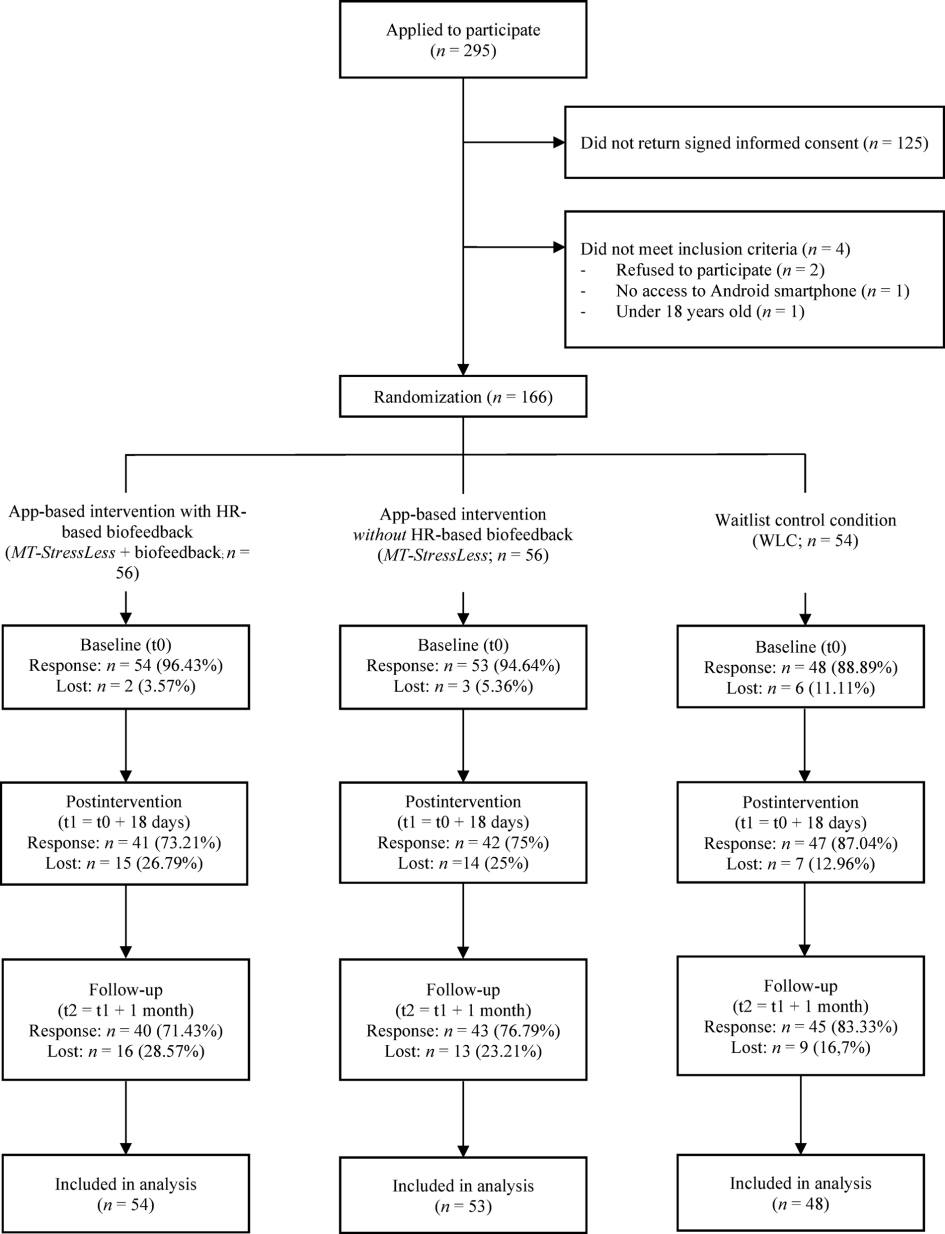
Participant flow, randomization, and response throughout the trial. HR = heart rate; *MT-StressLess* + biofeedback = Mentalis StressLess app-based intervention condition with heart rate-based biofeedback; *MT-StressLess* = Mentalis StressLess app-based intervention condition without heart rate-based biofeedback; WLC = waitlist control.

As shown in Fig. 1, a total of 295 individuals completed the initial screening questionnaire. Of these, 125 did not return the informed consent form, two explicitly refused to participate, one did not have a suitable smartphone, and one did not meet the minimum age threshold. Thus, a total 129 individuals were excluded from the study. The final sample of *N* = 166 participants was randomly allocated to one of the three study conditions via block randomization (block size of three, conducted by research staff not otherwise involved in the study via the methods outlined at https://randomizer.org/*).*

After randomization to the study conditions, participants were provided with a weblink to complete the baseline self-assessment (t0). Conducting the baseline assessment immediately after randomization ensured that the data accurately captured participants’ conditions immediately prior to the initiation of the intervention. Subsequently, participants in the two app-based intervention conditions received another weblink to download the *MT-Stressless* app, with instructions on how to install and use the app during the subsequent 18-day intervention period. Eighteen days later, at t1 all participants received another email containing a weblink to complete the assessment at postintervention (t0 + 18 days). After another four weeks at t2, all participants received a closing email requesting their online completion of the final follow-up assessment (t1 + one month). After the final follow-up assessment, participants in the WLC condition received access to the app without the HR-based biofeedback.

### Study conditions

See Table 1.

**Table 1:** Overview of intervention content across conditions.

Condition	Components	Details
*MT-StressLess*	14 stress management skills modules including: Psychoeducation Approach-Avoidance Modification Training Daily life skills practice	14 skills training modules: (1) progressive muscle relaxation (PMR)^10^ (2) cognitive restructuring of stress-enhancing thoughts^9^ (3) short version of PMR^10^ (4) time management^48^ (5) systematic explication and description of stress-inducing problems^49^ (6) goal setting^49^ (7) brainstorming methods^49^ (8) Identifying effective strategies, planning, and implementing them ^49^ (9) acceptance^50^ (10) ultra-short version of PMR^10^ (11) stress-reducing or compensating activities^51^ (12) mindfulness^52^ (13) sleep management^53^ (14) long-term prevention of stress^54^
*MT-StressLess* with heart rate (HR)-based biofeedback	All components of MT-StressLess and HR-based biofeedback component	HR-based biofeedback was implemented with the use of the smartphone’s built-in accelerometer. Participants practice stress induction and relaxation exercises, with real-time feedback on HR changes before AAMT tasks
WLC	No intervention during the study period Access to *MT-StressLess* (without biofeedback) after follow-up	Participants in the WLC condition complete assessments without receiving an active intervention. After the follow-up period, they are given access to the *MT-StressLess* app

*MT-StressLess* with HR-based biofeedback = Mentalis StressLess app-based intervention condition with heart rate-based biofeedback; *MT-StressLess* = Mentalis StressLess app-based intervention condition without heart rate based biofeedback; WLC = waitlist control.

### MT-StressLess

*MT-StressLess*, developed specifically for this study, is a fully automated app-based intervention designed to enhance stress management skills and reduce perceived stress. Conceptualized by MB, an expert in digital health interventions, the program is grounded in Lazarus and Folkman’s transactional model of stress^8^ and its distinction between problem-focused and emotion-focused coping. Although the content of the intervention was tailored for this study, similar approaches utilizing desktop-based Internet interventions grounded in this theoretical framework have demonstrated efficacy, particularly through the integration of problem-solving methodologies and emotion regulation techniques^55–57^. Table 1 provides an overview of the content.

#### Psychoeducation

Each module started with a psychoeducational component explaining the relevance of the respective skill and outlining methods for acquiring and fortifying it. To maximize participants’ engagement, this information was presented in the context of a fictional online group chat between four imaginary users sharing stress management difficulties, plus an E-Coach, all conversing on the nature of stress and how people might cope with high stress levels. Following principles originating from Socratic dialogue^58^, fictional conversations typically started with questions that were subsequently discussed and answered by the chat members and E-coach, respectively. The psychoeducational introduction concluded with a short quiz to assess participants’ comprehension of the information and subsequent feedback on their individual quiz performance.

#### Approach-avoidance modification training

The second component used principles of AAMT^59^ to elaborate on the content of the respective module in a presumably engaging manner. After a brief tutorial, participants were invited to swipe adaptive self-statement stimuli (e.g., “I will give my best to master my current chores, but I will also make sure that I give myself sufficient time to recharge my batteries afterward.”) downward-towards themselves, creating a zoom-in effect that conveyed a sense of reduced spatial distance. Contrastingly, they had to swipe dysfunctional self-statement stimuli (e.g., “I always have to do everything perfectly.”) upward-away from themselves, creating a zoom-out effect that generates the impression of increased spatial distance.

To further reinforce the game-like features of the intervention, our research team designed five novel modes for moving the AAMT stimuli on the smartphone screen: draw, plus-minus, select, command, and emotion recognition, in addition to the previously mentioned upward and downward swipe gestures (see Supplementary Materials, Table S2 for details). In the *emotion recognition steering* mode, the smartphone camera detected the user’s facial expressions. The sophisticated high-speed object recognition engine – SHORE© algorithm, a trained system utilizing annotated datasets of facial landmarks such as the eyes, nose, and mouth, analyzed these features to identify emotions^60^. This automated recognition of expressed emotions enabled dynamic interaction. For instance, positive emotions such as happiness (e.g., a smile) draw the stimulus closer to the user, with stronger expressions (e.g., a broad smile) resulting in faster movement. Conversely, negative emotions such as anger push the stimulus away, with the intensity of the emotion controlling movement speed. This interactive feedback loop allows users to adjust their facial expressions in real time based on stimulus movement, potentially increasing both user engagement and task accuracy. In addition, the calibration process ensures accurate emotion detection by accounting for individual baseline differences in neutral expressions.

The feasibility testing results on the software’s abitlity to recognize facial emotional expressions indicated that recognition could be impeded by interference from obscured faces or poor lighting conditions. Thus, as a backup option, participants were encouraged to use the *draw* or *plus-minus* modes. These modes, which involved either drawing directly on the screen or adjusting stimuli using plus and minus buttons, were unaffected by lighting conditions and provided a manual method for moving stimuli when algorithms failed to reliably recognize expressed emotions.

#### Skills practice in daily life

Each module’s third and final component required users to practice the respective skills by engaging in one or more tasks. The tasks involved completing skill-building exercises presented via text or audio files, recording answers to questions designed to prompt insight into effective stress coping, writing text messages to consolidate coping skills, creating lists of coping strategies, capturing photos of items relevant to stress coping, and carrying out coping-related actions (see Supplementary Materials, Table S3 for examples).

### User flow in *MT-StressLess*

Participants were encouraged to complete one module per day in the numerical sequence described above. It was recommended that participants pause their use of the app on weekends. Pausing app use on weekends was intended to encourage participants to apply the techniques in everyday situations, thereby facilitating the transfer of skills to real-life contexts^61^. Thus, adherence to these instructions resulted in an intervention period of 18 days in total (i.e., 14 weekdays of active app use and two sets of weekend days with no or less app use). This brief intervention duration was selected based on evidence suggesting that brief focused interventions can yield significant reductions in perceived stress^21^. Additionally, shorter intervention durations are associated with higher completion rates, as longer interventions require greater commitment from participants, which often increases the likelihood of dropout in app-based interventions^62^. To ensure that participants followed the recommended sequence of modules, access to subsequent training modules was granted only after meeting the following criteria: (a) completion of the final quiz of the psychoeducation section of each previous module, (b) completion of at least one AAMT task of the previous module, and (c) completion of at least three skill practices in daily life tasks from the previous module. Participants could practice a novel module as often as desired when it was made available. To encourage high engagement with the app and promote intervention adherence, participants could tap buttons on their home screens to access general information on stress and coping strategies. This beneficial feature of providing participants with a personal dashboard to illustrate the immediate effects of utilizing the intervention was further optimized by providing motivational feedback aligned with the displayed effects.

### MT-StressLess with HR-based Biofeedback

In the *MT-StressLess* with HR-based biofeedback condition, participants used an augmented version of the basic *MT-StressLess* intervention enhanced with an HR-based biofeedback component, but otherwise identical to the app without the biofeedback component. This component targeted the interaction between stress, indicated by an elevated HR induced through imagining a stressful scenario, and a subsequent relaxation exercise designed to reduce stress.

Anticipating a stressful event can activate the sympathetic nervous system, which leads to an increase in HR as part of the “fight or flight” response. This process triggers the release of stress hormones such as adrenaline, priming the body for immediate action. Notably, anticipating stress can elicit physiological responses similar to those experienced during actual stress, including elevated HR and blood pressure^63^. Once stress is identified, biofeedback applications can guide users through targeted relaxation techniques such as deep breathing or guided imagery to downregulate the physiological stress response. Over time, repeated practice helps users strengthen their interoception, the ability to recognize internal bodily sensations, and develop better stress-coping skills^29^.

In the intervention *MT-StressLess* with HR-based Biofeedback, HR was measured via ballistocardiography^64^ using the smartphone’s acceleration sensors to detect the physical movement generated by the heartbeat^65^. From these data, an HR indicator was extracted in near real time using an algorithm tailored specifically for this purpose by Global Vitals LLC. The accelerometer data were processed by applying a moving average filter to remove baseline drift, followed by standardization of the components for orientation consistency. A band-pass Butterworth filter was used to isolate the ballistocardiogram movements, and the final pulse waveform was refined using another filter. Fast Fourier Transform analysis was then used to identify the HR by locating the frequency peak between 0.66 and 2.5 Hz, corresponding to 45–150 beats per minute (bpm)^66,67^. Infeasibility analyses conducted prior to the start of the study, we determined that a decrease of 10 bpm provided the most accurate classification of the transition from stress to relaxation, thereby ensuring a robust distinction between the two states. Additionally, a secondary threshold, a 2 bpm reduction from baseline, was introduced to account for participants who did not exhibit a pronounced HR increase during the stress phase but still demonstrated a measurable decline during relaxation.

**Fig. 2:**
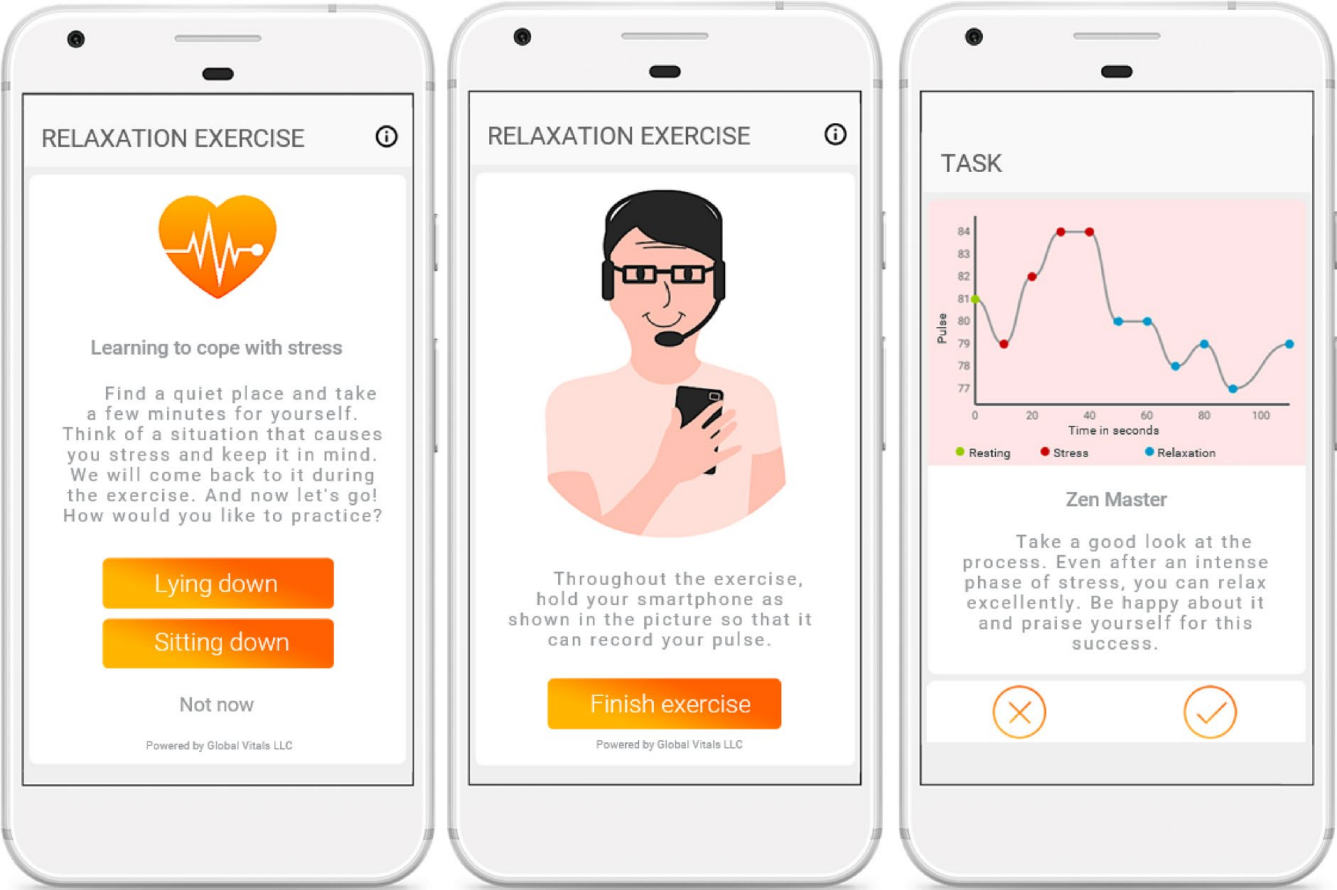
Screenshots of the instruction and feedback of the relaxation exercise. *MT-StressLess* with HR-based biofeedback condition included a relaxation exercise. Participants were asked to identify a typical stress situation and to choose a body posture for the exercise (left). They were then asked to hold their smartphone near their heart during the audio instruction (middle). Participants were then shown their HR during the relaxation exercise and given motivational feedback, depending on how the HR decreased during the relaxation phase (right).

For the main trial, the HR-based biofeedback component was offered exclusively to participants in the HR-based biofeedback condition prior to each AAMT task. Users could repeat the exercise (i.e., practice) as often as they wanted or skip it if they felt it was not an appropriate time or place to engage in it. At the beginning of each biofeedback exercise, users were instructed to adopt a sitting or lying position in a quiet place and to mentally identify a stressful situation of current significance. They were then asked to follow audio-based instructions guiding them to: (1) place the smartphone on the left side of their chest over their heart, (2) rest for 10 s (s) (baseline-HR assessment), (3) think of the previously identified stressful situation for 40 s (stress-HR assessment), and (4) apply muscle and breathing relaxation in accordance with further instructions that were based on the user’s HR (HR assessment during relaxation). If HR was 10 bpm below the maximum value measured during the “stress phase” or 2 bpm less than the maximum value at baseline, a reinforcing audio feedback (“Very good!”) was played. If HR did not meet one of these criteria within the first 40 s of the exercise, audio instructions on muscle and breathing relaxation were continued for another 20 s. If neither the HR decrease criterion was attained during this 20 s period, the instructions were continued for a second time for another 20 s. As soon as one of the HR decrease criteria was reached or the third repetition of the audio instructions was completed, further audio instructions guided participants through a gradual disengagement from the relaxation exercise and a transition to the first AAMT task of the respective module. From this point onward, participants had access to feedback on the course of their HR during the exercise (with comments varying according to the individual course; see Fig. 2 for an example). Furthermore, in the *MT-StressLess* with HR-based biofeedback condition, participants were instructed to look at the graph representation of their HR during the biofeedback exercise and praise themselves for any success they achieved in decreasing their HR (or for their effort in trying) during the first task of the respective module.

### Waitlist control condition

In the WLC condition, participants completed assessments only and were given access to the *MT-StressLess* intervention after follow-up.

### Measures

#### Adherence and usability information

In both active conditions, app usage data were recorded automatically, including time spent in the app, number of usage days, completed modules, solved psychoeducation quizzes, solved AAMT tasks, solved skill-practice tasks in daily life, and engagement with the HR biofeedback exercise.

To assess usability feedback, participants in both active conditions completed the system usability scale (SUS; German version: Rummel, 2016^68^) at postintervention. The usability score ranges from 0 to 100, with higher scores reflecting greater perceived usability. Furthermore, participants were asked to respond to three self-developed questions assessing comprehensibility (e.g., “The explanations in the chat format were easy to understand”), appeal (e.g., “The presentation in the chat format was appealing”), and goal achievement (e.g., “The explanations in the chat format were useful in achieving my goal (knowledge acquisition)”) in relation to the specific content of the intervention, which included psychoeducation, quizzes, AAMT tasks, daily life tasks, and the HR biofeedback exercise. Responses were rated on a scale ranging from 1 (*strongly disagree*) to 5 (*strongly agree*).

#### Primary outcome

The primary outcome measure of this study was the Perceived Stress Scale-10 (PSS-10; German Version: Klein and colleagues, 2016^69^). This self-report instrument assesses the degree of perceived stress using ten items (e.g., “In the last month, how often have you felt nervous and stressed?”) to be rated on a five-point Likert scale (ranging from 0 = *never* to 4 = *very often*).

#### Secondary outcomes

We used the Emotion Regulation Skills Questionnaire (ERSQ-27; German version: Berking & Znoj, 2008^70^) to assess the successful application of adaptive emotion regulation skills (arguably, an important component of coping with stress). Higher average scores indicate greater use of adaptive emotion regulation skills. To assess the severity of depressive symptoms, we used the Patient Health Questionnaire (PHQ-9; German version: Martin and colleagues, 2006^71^). Higher scores indicate more severe symptoms of depression. The WHO-5 Well-Being Index (WHO-5; German version: Brähler and colleagues, 2007^72^) was used to assess subjective well-being. Lower values indicate poorer well-being. For a more detailed description of all measures, please see Supplemental Material.

### Data analysis

To identify differences in demographic data and clinical characteristics between the study conditions, we used χ^2^ tests, analyses of variance (ANOVAs), and corresponding nonparametric tests. Adherence and usability data were reported descriptively and compared between groups using χ^2^ and Wilcoxon rank-sum tests (IBM SPSS 26; IBM Corp, Armonk, NY, USA). All randomized participants with available baseline values were included in further analyses in accordance with the intention-to-treat (ITT) principle.

To examine changes in the primary (perceived stress) and secondary outcomes (emotion regulation skills, depressive symptoms, and overall well-being) over time across the three study conditions, Linear mixed-effects models (LMMs) were employed (*lme4* package^73^ in R (version 4.4.2)). LMMs are particularly well-suited for the analysis of longitudinal data with repeated measures, as they appropriately account for the intrinsic dependencies within such data structures^74^. Moreover, LMMs yield robust and reliable estimates even in the presence of missing data by employing maximum likelihood estimation, which ensures the generation of unbiased results under the Missing at Random assumption^75^. The LMMs incorporated the following fixed effects: condition (*MT-StressLess* with HR-based biofeedback, *MT-StressLess* alone, and WLC, with the respective reference category depending on the respective model), time point (baseline, postintervention, and follow-up), and their interaction. Random intercepts for participants (id) were included to account for within-subject correlations across repeated measures. The models were fitted using restricted maximum likelihood, and Satterthwaite’s approximation was used to compute the degrees of freedom for significance testing. To assess the significance of fixed effects, we first conducted a Type-III ANOVA within the LMM framework^76^. The condition variable was coded as a factor, using the WLC condition as the reference category in the first step and the *MT-StressLess* alone condition in the second step. Similarly, time was coded as a factor with baseline set as the reference category. Between-condition effect sizes (Cohen’s *d*) were calculated by dividing the estimated mean difference at postintervention or follow-up by the pooled standard deviations of the observed means at baseline^77^ and were interpreted as small (0.20), medium (0.50), and large (0.80).

Furthermore, to examine associations between app usage and postintervention outcomes, we first computed Spearman’s rank correlation coefficients to assess relationships between app usage metrics (i.e., minutes spent in the app, active usage days, solved AAMT tasks, and, in the *MT-StressLess* with HR-based biofeedback condition, HR biofeedback tasks successfully completed by achieving the predefined relaxation state) and postintervention PSS scores, residualized by baseline values. We chose this approach because LMMs lacked sufficient power for reliable estimates. Since correlation analyses only capture the strength and direction of associations without modeling predictive relationships, we conducted separate linear regression analyses within each intervention condition. These models allowed us to examine the extent to which app usage predicted baseline-adjusted postintervention PSS scores while accounting for potential confounding effects. To address potential inflation of Type I error due to multiple comparisons and conceptual overlap between predictors, we applied a Benjamini–Hochberg correction^78^ to the correlation analyses and the p-values of individual predictors in the regression models. We set the critical significance level alpha to 0.05.

## Results

### Baseline demographics

Neither demographic variables nor the average perceived stress (PSS-10) sum scores differed across study conditions at the initial screening (all *ps* ≥ 0.097, see Table 2). Despite efforts to minimize study attrition (e.g., by means of up to three email reminders), 36 of the 166 participants (21.69%) were lost at postintervention assessment and 38 participants (22.89%) did not complete the follow-up assessment. On average, participants lost to postintervention were older (*M* age = 27.17, *SD* = 9.84) than those who remained at postintervention (*M* age = 23.3, *SD* = 7.20, t (45.89) = 2.2, *p* = 0.033) but did not differ with regard to any other variable included in Table 2 (all *p*s ≥ 0.220, for detailed statistics, see Supplemental Material, Table S4, S5). Additional analyses showed no significant differences in drop-out rates between study conditions at postintervention (χ^2^ (2, *N* = 166) = 3.64, *p* = 0.162, *V* = 0.15) or at follow-up (χ^2^ (2, *N* = 166) = 2.12, *p* = 0.330, *V* = 0.12). Further analyses of the final sample indicated that the rate of missing data was below 25% and that missing data were completely at random, MCAR Test (χ^2^ = 34.53, *df* = 30, *p* = 0.260). Thus, the conditions for the use of LMMs^75^ were met.

**Table 2:** Sociodemographic and Clinical Characteristics of Participants at Screening.

Variable	*MT-StressLess* + biofeedback (*n* = 56)		*MT-StressLess* (*n* = 56)		WLC (*n* = 54)		Statistics			
							*df*	*x*^2^*/F*	*p*	*R*^2^*/V*
Gender							4	7.48	0.112	0.15
Female (*n*, %)	38	67.86	49	87.5	42	77.77				
Male (*n*, %)	17	30.36	7	12.50	12	22.22				
Diverse (*n*, %)	1	1.79	0	0	0	0				
Age (*M, SD*)	25.39	9.88	23.25	7.27	23.76	6.29	2; 163	1.10	0.335	0.001
Age (range)	18–60		18–59		18–60					
Highest education degree							10	11.13	0.348	0.18
None (*n,* %)	1	1.79	0	0	0	0				
Secondary General School (*n,* %)	1	1.79	1	1.79	0	0				
Intermediate Secondary School (*n,* %)	4	7.14	2	3.57	0	0				
Graduate (*n,* %)	38	67.86	40	71.43	33	61.11				
Bachelor or Master Degree (*n,* %)	11	19.64	12	21.42	20	37				
PhD (*n,* %)	1	1.79	1	1.79	1	1.85				
Employement							6	5.54	0.477	0.13
Employed (*n,* %)	4	7.14	7	12.50	2	3.70				
Unemployed (*n,* %)	2	3.57	0	0	2	3.70				
Student (*n,* %)	41	73.21	37	66.07	40	74.07				
Others (*n,* %)	9	16.07	12	21.42	10	18.52				
Health-related variables										
Current psychological/psychiatric treatment (*n,* %)	4	7.14	5	8.93	4	7.41	Fisher’s exact test		0.859	
Current cardiovascular disease (*n,* %)	3	5.36	3	5.36	1	1.85	Fisher’s exact test		0.701	
Currently smoking cessation (*n,* %)	6	10.71	1	1.79	6	11.11	Fisher’s exact test		0.097	
Physical activity per week, in hours (*M, SD*)	3.90	3.02	3.63	3.72	3.31	3.69	2; 157	0.38	0.687	-0.008
Experience in relaxation exercises (*n,* %)	35	62.50	37	66.07	29	53.70	2	1.86	0.394	0.11
Frequency of smartphone use in daily life (*M, SD*)	4.12	0.95	4.25	0.72	4.19	0.80	2; 163	0.22	0.729	-0.008
PSS-10 at screening (*M, SD*)	21.05	6.84	23.30	6.49	22.37	6.94	2; 163	1.57	0.212	0.007

*MT-StressLess* + biofeedback = Mentalis StressLess app-based intervention condition with heart rate-based biofeedback; *MT-StressLess* = Mentalis StressLess app-based intervention condition without heart rate based biofeedback; WLC = waitlist control; Frequency of smartphone use was assed via the self-developed question “How often do you use your smartphone?”; item range: 1 (very rarely) – 5 (very often); PSS-10 = Perceived Stress Scale.

### Intervention adherence and usability

Regarding app usage, 11 participants (9.82%) did not download the app, and 5 (4.46%) did not engage with at least one competence. On average, participants completed *M* = 7.45 competencies (53.21%), with 25 participants (26.04%) completing all 14 competencies and 51 (53.12%) completing at least seven. Chi-square and Wilcoxon rank-sum tests showed no significant differences between conditions. Further analyses revealed no differences in (a) active usage days, (b) minutes spent in the app, (c) completed psychoeducation quizzes, (d) solved AAMT tasks, or (e) solved tasks (all *p*s > 0.085; see Supplemental Material, Table S6). In the *MT-StressLess* with HR-based biofeedback condition, 73% of the participants used the HR biofeedback exercise at least once. On average, these 36 individuals started 8.78 relaxation exercises, resulting in 316 exercises, of which 87.66% were completed. Of the 277 exercises completed, 67.87% achieved the predefined relaxation state, while 32.13% did not.

Regarding usability, the overall SUS score was *M* = 83.51, *SD* = 11.95 out of 100, which is considered good-to-excellent usability^79^. A Wilcoxon rank-sum test revealed a significant difference between conditions (*W* = 1246, *p* < 0.001), with *MT-StressLess* with HR-based biofeedback (*M* = 87.38, *SD* = 8.94) scoring higher than *MT-StressLess* (*M* = 79.29, *SD* = 13.55). Participants rated the psychoeducation, quizzes, AAMT tasks, and daily-life tasks in terms of comprehensibility, appeal, and goal achievement. Comprehensibility was rated high across all components (*M* = 4.28–4.85, *SD* = 0.46–1.04). Appeal varied, with daily-life tasks receiving higher scores (*M* = 4.24, *SD* = 0.98) while quizzes (*M* = 3.21, *SD* = 1.24) and AAMT tasks (*M* = 2.73–3.11, *SD* = 1.03–1.45) were rated lower. Goal achievement ratings ranged from (*M* = 3.54–4.28, *SD* = 0.66–1.20) with AAMT tasks receiving the lowest scores. Participants’ perceptions of the biofeedback-based relaxation exercise were mixed. While the task was rated as highly comprehensible (*M* = 4.28, *SD* = 1.04) its appeal (*M* = 3.35, *SD* = 1.41) and perceived goal achievement (*M* = 2.85, *SD* = 1.19) were lower; see Supplemental Material, Tables S7–S9 for details.

### Intervention effects on primary outcome

**Fig. 3:**
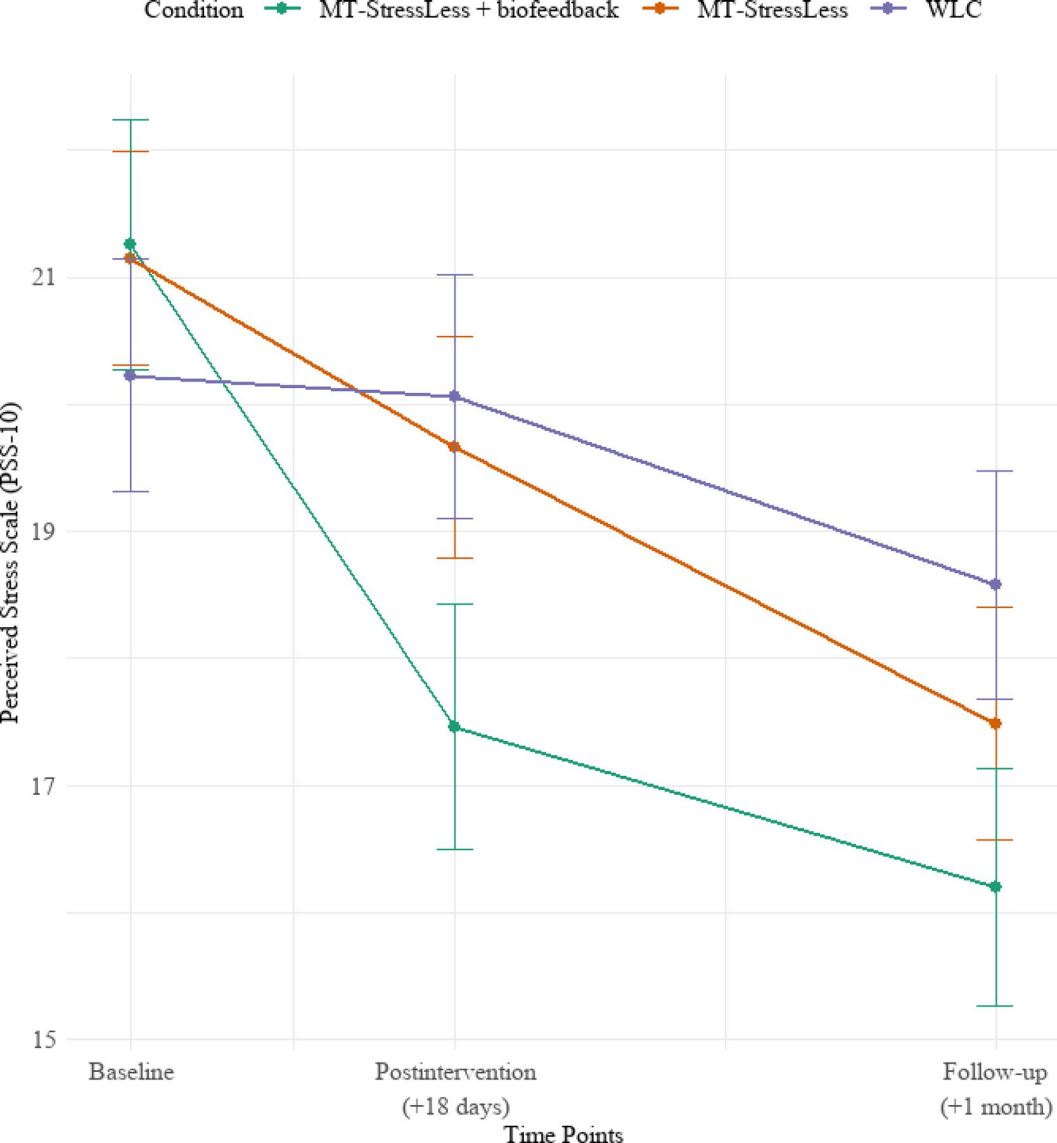
Observed means of the Perceived Stress Scale (PSS-10) across time points (Baseline, Postintervention, and Follow-up) for each condition (MT-StressLess + biofeedback, MT-StressLess, and WLC). Error bars represent ± 1 standard error of the mean (SE).

Figure 3 presents the observed means for the primary outcome, PSS, across the three conditions, *MT-StressLess* with HR-based biofeedback, *MT-StressLess*, and WLC, measured at baseline, postintervention, and at the 4-week follow-up (see Supplemental Material, Table S10). Table 3 provides the estimated means and overall effects (Time × Condition interaction) for PSS across the three conditions and three assessment time points. Table 4 displays the condition comparisons and between-condition effect sizes. A linear mixed model revealed a significant Time × Condition interaction for the primary outcome PSS (*F*(4, 255.43) = 3.25, *p* = 0.013) (see Supplemental Material, Tables S11, S12 for detailed results). To address the primary aim, pairwise comparisons within the LMM revealed that the *MT-StressLess* with HR-based biofeedback condition demonstrated a significantly greater reduction in PSS compared to the WLC condition, at postintervention (*t*(257.52) = -3.27, *p* = 0.001, *d* = 0.41, 95% CI [0.03, 0.79]) and at follow-up (*t*(258.13) = −2.77, *p* = 0.006, *d* = 0.55, 95% CI [0.17, 0.93]). In further exploratory analyses, the *MT-StressLess* without HR-based biofeedback condition did not differ significantly from the WLC condition, at postintervention, (*t*(255.60) =  −1.41, *p* = 0.161, *d* = 0.14, 95% CI [-0.24, 0.51]) or at follow-up, (*t*(255.77) = −1.78, *p* = 0.076, *d* = 0.44, 95% CI [0.06, 0.82]). Similarly, when directly comparing the two active intervention conditions, no significant difference was observed either at postintervention (*t*(263.43) =  −1.84, *p* = 0.068, *d* = 0.29, 95% CI [−0.08, 0.66]) or at follow-up (*t*(263.45) = −1.00, *p* = 0.317, *d* = 0.15, 95% CI [−0.22, 0.52]).

**Table 3:** Estimated Means for Primary and Secondary Outcome Measures and Overall Effects.

Outcome	*MT-StressLess* + biofeedback (*n* = 54)		*MT-StressLess* (*n* = 53)		WLC (*n* = 48)		Overall effects (Time × Condition interaction)		
	*M*	*SE*	*M*	*SE*	*M*	*SE*	*df*	*F*	*p*
PSS-10							4, 255.43	3.25	0.011
Baseline	21.26	0.87	21.15	0.88	20.23	0.92			
Post	17.44	0.94	19.38	0.94	19.99	0.93			
Follow-up	16.22	0.95	17.23	0.93	18.26	0.94			
ERSQ-27							4, 259.96	4.95	< 0.001
Baseline	59.80	2.18	63.74	2.20	62.33	2.31			
Post	72.30	2.37	72.66	2.36	63.41	2.32			
Follow-up	70.99	2.39	71.16	2.34	63.86	2.35			
PHQ-9							4, 257.96	1.56	0.185
Baseline	9.91	0.64	9.19	0.64	8.75	0.67			
Post	7.84	0.69	8.33	0.68	8.36	0.68			
Follow-up	7.83	0.69	7.07	0.68	7.64	0.68			
WHO-5							4, 259.96	3.10	0.016
Baseline	10.39	0.62	10.94	0.62	11.33	0.65			
Post	12.23	0.67	12.17	0.66	11.04	0.66			
Follow-up	12.69	0.67	12.96	0.66	11.43	0.66			

*MT-StressLess* + biofeedback = Mentalis StressLess app-based intervention condition with heart rate-based biofeedback; *MT-StressLess* = Mentalis StressLess app-based intervention condition without heart rate-based biofeedback; WLC = waitlist control; PSS-10 = Perceived Stress Scale; ERSQ-27 = Emotion Regulation Skills Questionnaire; PHQ-9 = Patient Health Questionnaire; WHO-5 = WHO-Five Well-Being Index.

**Table 4:** Pairwise Condition Comparisons and Effect Sizes.

Outcome and conditions	Post					Follow-up				
	*df*	*t*	*p*	*d*	95% CI	*df*	*t*	*p*	*d*	95% CI
PSS-10										
*MT-StressLess* + biofeedback vs WLC	257.52	−3.27	0.001	0.41	[0.03, 0.79]	258.13	−2.77	0.006	0.55	[0.17, 0.93]
*MT-StressLess* vs WLC	255.60	−1.41	0.161	0.14	[−0.24, 0.51]	255.77	−1.78	0.076	0.44	[0.06, 0.82]
*MT-StressLess* + biofeedback vs *MT-StressLess*	263.43	−1.84	0.068	0.29	[−0.08, 0.66]	263.45	−1	0.317	0.15	[−0.22, 0.52]
ERSQ-27										
*MT-StressLess* + biofeedback vs WLC	262.09	4.03	< 0.001	−0.58	[−0.96, −0.20]	262.76	3.37	< 0.001	−0.47	[−0.85, 0.09]
*MT-StressLess* vs WLC	260.10	2.78	0.006	−0.59	[−0.97, −0.20]	260.29	2.08	0.031	−0.46	[−0.84, 0.08]
*MT-StressLess* + biofeedback vs *MT-StressLess*	268.27	1.24	0.218	−0.02	[−0.39, 0.35]	268.29	1.31	0.193	−0.01	[−0.38, −0.36]
WHO-5										
*MT-StressLess* + biofeedback vs WLC	261.99	2.80	0.006	−0.25	[−0.62, 0.13]	262.57	2.84	0.005	−0.26	[−0.64, 0.11]
*MT-StressLess* vs WLC	260.14	2.00	0.043	−0.27	[−0.65, 0.11]	260.30	2.51	0.012	−0.37	[−0.74, 0.01]
*MT-StressLess* + biofeedback vs *MT-StressLess*	267.65	0.79	0.430	−0.01	[−0.38, 0.36]	267.65	0.37	0.713	−0.06	[−0.43, 0.31]

### Sensitivity analyses

To assess the robustness of the results for the primary outcome, we conducted sensitivity analyses using the per-protocol sample, including only participants who actively engaged with the intervention, and a model controlling for sex and age. The findings from both the per-protocol analysis and the model controlling for sex and age were consistent with those of the main analyses. Detailed results of these sensitivity analyses are provided in the Supplementary Materials, Tables S13–S16.

### Intervention effects on secondary outcomes

Table 3 provides the estimated means and overall effects (Time × Condition interaction) for the secondary outcomes. Table 4 displays the condition comparisons and between-condition effect sizes. Significant Time × Condition interactions were found for emotion regulation skills (*F*(4, 259.96) = 4.95, *p* < 0.001) and well-being (*F*(4, 259.96) = 3.10, *p* = 0.016) but not for depressive symptoms (*F*(4, 257.96) = 1.56, *p* = 0.185) (see Supplemental Material, Tables S17–S20 for detailed results). Pairwise comparisons within the LMM showed that both active intervention conditions significantly improved emotion regulation skills at postintervention (*MT-StressLess* with HR-based biofeedback: *d* = −0.58, *p* < 0.001; *MT-StressLess*: *d* = −0.59, *p* = 0.006) and at follow-up (*d* = −0.47, *p* < 0.001; *d* = −0.46,* p* = 0.031), as well as well-being at postintervention (*d* = −0.25, *p* = 0.006; *d* = −0.27, *p* = 0.043) and follow-up (*d* = −0.26, *p* = 0.005; *d* = −0.37, *p* = 0.012), compared to the WLC condition. No significant differences were found between the two active conditions for either outcome (emotion regulation skills: *p* > 0.193; well-being: *p* > 0.430).

### App usage effects

Spearman’s rank correlations revealed that higher app engagement was associated with lower postintervention perceived stress adjusted for baseline values. In the *MT-StressLess* condition, more minutes spent in the app (*r*_s_ = −0.41, *p* = 0.025) and a greater number of completed AAMT tasks (*r*_s_ = −0.36, *p* = 0.038) were significantly related to reduced stress levels. In the *MT-StressLess* with HR-based biofeedback condition, no statistically significant negative associations were found; however, similarly strong correlations emerged. Notably, there was a trend towards a negative association for successfully completed HR biofeedback tasks (*r*_s_ = −0.35, *p* = 0.073). Further details are provided in Supplemental Material, Table S6. We conducted consecutive separate linear regression analyses to assess the predictive value of app usage on postintervention stress levels. In both conditions, minutes spent in the app were a significant predictor of postintervention PSS scores (*MT-StressLess*: β = −0.01, *p* < 0.001, R^2^ = 14.97%; *MT-StressLess* with HR-based biofeedback: β = −0.02, *p* < 0.001, R^2^ = 9.92%). A similar pattern emerged for the number of solved AAMT tasks, which was also significantly associated with PSS scores (*MT-StressLess*: β = −0.24, *p* = 0.003, R^2^ = 8.42%; *MT-StressLess* with HR-based biofeedback: β = −0.25, *p* = 0.003, R^2^ = 7.15%). Successfully achieving the predefined relaxation state during HR biofeedback tasks was a significant predictor of postintervention PSS scores (β = −0.50, *p* < 0.001), explaining 15.95% of the variance.

## Discussion

This study aimed to evaluate the efficacy of a brief and novel smartphone-based app for stress management that included an accelerometer-derived HR-based biofeedback component. To this end, we tested the hypothesis that the new intervention would be superior to a WLC condition in terms of perceived stress as well as in improving secondary outcomes, including emotion regulation skills, depressive symptoms, and well-being. In exploratory analyses, we examined whether a non-biofeedback version of the intervention was also superior to the WLC condition, and whether the intervention with HR-based biofeedback was more effective than the otherwise identical non-biofeedback version. Finally, we assessed usability feedback and explored the impact of app usage on the intervention outcomes.

Results from our three-arm RCT (N = 166) indicated that participants receiving the *MT-StressLess* intervention with HR-based biofeedback reported significantly greater reductions in perceived stress than those in the WLC condition. These effects were maintained at the 1-month follow-up and remained consistent across multiple sensitivity analyses. In exploratory analyses, participants in the *MT-StressLess* condition without biofeedback showed small reductions in perceived stress that were not significantly different from those in either of the other conditions.

The between-condition effect size for the *MT-StressLess* with HR-based biofeedback intervention on perceived stress at postintervention was *d* = 0.41. To our knowledge, this is the first study to combine a smartphone-based stress management intervention with accelerometer-derived HR biofeedback, thus precluding comparisons with similar studies. However, the effect size found in this study exceeded the average effect size for stress reduction in smartphone-based interventions reported in a recent meta-analysis (*g* = 0.29)^80^ and yielded more favorable results than those reported in the study by Yoon and colleagues, which used using smartphone-camera-based photoplethysmography to measure HRV^39^. This finding, along with the favorable results for our secondary outcomes emotion regulation, and well-being (but not depressive symptom severity), underscores the potential of sensor-, and particularly accelerometer-based technologies for HR biofeedback in combination with a stress management intervention to improve stress.

With regard to the main finding, that *MT-StressLess* with HR-based biofeedback outperformed the WLC condition, one plausible mechanism is that the intervention with integrated HR-based biofeedback may have enhanced stress regulation by increasing interoceptive awareness and improving autonomic balance beyond the immediate effects. The literature shows that real-time HR biofeedback helps individuals better detect physiological stress signals (e.g., elevated HR) and apply relaxation techniques, such as slow-paced breathing, to activate the parasympathetic nervous system^81^. Furthermore, biofeedback may indirectly facilitate prefrontal cortex regulation of the amygdala, thereby reducing emotional reactivity and enhancing top-down stress control^82^. These mechanisms align with prior research showing that biofeedback enhances self-regulation, emotional awareness, and autonomic flexibility, contributing to sustained stress reduction^34,83,84^. In addition to HR as an instant feedback measure, HRV provides a more reliable indicator of long-term stress regulation capacity as it is associated with better emotion regulation, greater stress resilience, and reduced physiological arousal^16,85^. However, HRV requires longer measurement periods to ensure accuracy^86^ than what we could implemented here. Future studies should integrate continuous HRV measurements to better assess overall stress resilience and the lasting effects of biofeedback interventions.

While these mechanisms may partly explain the superiority of the biofeedback-enhanced intervention over the WLC condition, the exploratory finding that the core intervention neither significantly reduced perceived stress compared with the WLC condition nor differed significantly from the biofeedback-enhanced condition suggests that both the efficacy of the core intervention and the added benefit of the biofeedback component remain uncertain. This underscores the need for cautious interpretation and highlights the potential role of other specific and non-specific factors.

One possible factor is limited statistical power. Although it is possible that both the comparison between *MT-StressLess* alone and the WLC condition, as well as the comparison between the core and the biofeedback-enhanced intervention, might have reached statistical significance with a larger sample size, it remains questionable whether such differences would also indicate clinically relevant change. To achieve such change, the core intervention may need to offer a longer duration or greater intensity, as the relatively brief format may not have provided sufficient time for participants to internalize and consistently apply the provided strategies. The limited evidence of an added benefit, may be related to how the biofeedback component was operationalized, particularly the use of static HR cutoffs derived from a small healthy sample to define stress-to-relaxation transitions. Individual differences in baseline HR, cardiovascular fitness, and stress responsiveness may have limited the sensitivity of this approach. Future studies should consider adaptive or individualized thresholds to enhance accuracy and engagement. Furthermore, adherence and usability seemed to play a crucial role, as only 26.04% of participants completed all 14 competencies and 21.69% of participants dropped out at postintervention. While these figures are in line with previous smartphone-based interventions, they underscore the need for improved retention strategies^87,88^. Usability data suggest that the intervention was user-friendly and well-structured, with high comprehensibility. However, AAMT tasks, quizzes, and the biofeedback relaxation exercise had lower appeal and goal attainment ratings, indicating room for improvement. Additionally, app usage appeared to contribute to the intervention effects. In both active conditions, greater engagement was associated with lower perceived stress at postintervention. This pattern was particularly noticeable in the *MT-StressLess* with HR-based biofeedback condition, in which a higher number of completed biofeedback relaxation tasks tended to be associated with greater stress reduction. Digital interventions rely on self-guided engagement^89^, and without immediate reinforcement, some participants may have had difficulty maintaining motivation or consistently applying stress management techniques. However, because engagement metrics (e.g., time spent in the app and active usage days) did not differ significantly between conditions, biofeedback’s primary contribution may have been in enhancing perceived efficacy rather than overall usage levels. Another important factor to consider is the placebo or outcome expectancy effect. Participants in the *MT-StressLess* with HR-based biofeedback condition may have expected greater benefits due to the novelty and perceived sophistication of the biofeedback component. This expectation may have led to increased motivation, engagement, and subjective stress reduction. By contrast, participants in the *MT-StressLess*-only condition may not have had the same level of expectation, potentially influencing their perceived benefits. However, expectancy effects also contribute to the efficacy of face-to-face mental health interventions^90^, as widely acknowledged in common factor models of psychotherapy^91^, and have also been discussed as a potential mechanism of action in smartphone-based interventions^92^. Thus, expectancy is a legitimate, yet insufficiently understood factor that should be considered as part of an intervention and further explored as a potential therapeutic target. It is likely that, in our study, a combination of specific intervention benefits and common factors, such as expectation effects, contributed to the greater perceived stress reduction observed in the *MT-StressLess* condition with HR-based biofeedback.

This study offers several strengths, particularly the innovative integration of smartphone-based HR biofeedback using built-in sensors, eliminating the need for external devices, and enhancing accessibility and scalability. The rigorous three-arm RCT design allowed an exploratory evaluation of the standalone intervention as well as the added benefit of biofeedback, while LMMs ensured robust statistical analysis. Despite its strengths, this study has several limitations. First, both the *MT-StressLess* core intervention and the biofeedback component were novel interventions. The absence of an established state-of-the-art active control group restricts direct comparisons with established stress management programs. This limits the ability to draw strong conclusions about the efficacy of the *MT-StressLess* with HR-based biofeedback intervention relative to existing digital or other (established) interventions. Additionally, because no condition specifically emphasized relaxation without HR-based biofeedback, it remains unclear whether the observed effects were due to the biofeedback itself or general relaxation techniques. Future research should compare HR-based biofeedback with other interventions and alternative relaxation methods, to isolate their unique contributions. Second, although the sample size was relatively large, it was still too small to detect more subtle intervention effects, allow for sophisticated moderation and mediation analyses, or enable a thorough investigation of usage patterns (e.g., time spent on individual modules and adherence to components) to better understand the relationship between app engagement and intervention efficacy. Moreover, the self-selected sample was skewed toward younger female Android users, limiting generalizability. Future studies should aim for larger, more diverse samples to improve external validity. Third, the exclusive reliance on self-reported data introduces potential placebo, expectancy, and social desirability effects, which may have influenced the results^93^. This is a common challenge in clinical research, where participants may feel inclined to provide responses that reflect positive change rather than fully objective assessments of their experiences^93^. Thus, future studies should complement self-reports with physiological and behavioral indicators of stress, such as longitudinal HRV measurements^94^. Finally, the one-month follow-up period in the present study was relatively brief compared to app-based targeting mental disorders^95,96^. However, when compared with analogous stress intervention studies, which often lack a follow-up assessment point^22,97^, it represents a methodological advancement. Nevertheless, the stability of initial effects over longer periods remains unknown and should be evaluated in future studies.

In conclusion, the findings point to the potential of combining smartphone-based interventions with HR-based biofeedback to reduce perceived stress. However, non-specific factors such as placebo effects, outcome expectancy, and user engagement, as well as the limited efficacy of both the core intervention and the biofeedback component in their current form, may also have influenced the observed outcomes. These findings highlight the need to better understand optimal intervention duration, motivation, reinforcement, and more individualized approaches to stress reactivity. While the novel and practical HR-based biofeedback approach, the three-arm RCT design, the statistical modeling, and objective app usage tracking strengthened the study’s findings, certain limitations should be acknowledged, including the lack of a state-of-the-art active control condition and a standalone HR-based biofeedback condition, the sample size and characteristics restricting some insights into mechanisms of change, the reliance on self-reported data, the chosen static HR cutoffs, and a brief follow-up period of one month. Although data collection took place in 2017, sensor-based interventions remain in their early stages, and research in this field continues to advance. Cutting-edge technologies, such as machine learning and artificial intelligence (AI), have the potential to enhance personalization and real-time feedback^23^; yet the integration of smartphone-based physiological sensing remains an evolving area. This study provides a valuable foundation for future research exploring how advancements in machine learning, AI-driven personalization, and sensor technology can further optimize real-world applications while carefully considering ethical aspects as sensor-based interventions continue to evolve.

## Electronic supplementary material

Below is the link to the electronic supplementary material.

Supplementary Material 1

## Data Availability

All data have been made publicly available at the Open Science Framework (OSF) and can be accessed at https://osf.io/6rfm5/?view_only=64e72b8a85a74b5bba460c39bf9ed5a7.

## References

[1] Li, R. et al. The influence of perceived stress and income on mental health in China and Germany. *Heliyon*10.1016/j.heliyon.2023.e17344 (2023). 37408921 10.1016/j.heliyon.2023.e17344 PMC10318459

[2] Association, A. P. *Stress in America 2022: Concerned for the future, beset by inflation*, https://www.apa.org/news/press/releases/stress/2022/concerned-future-inflation (2022).

[3] Ray, J. *World Unhappier, More Stressed Out Than Ever*, https://news.gallup.com/poll/394025/world-unhappier-stressed-ever.aspx (2022).

[4] O’Connor, D. B., Thayer, J. F. & Vedhara, K. Stress and health: A review of Psychobiological processes. *Annu. Rev. Psychol.* **72**, 663–688. 10.1146/annurev-psych-062520-122331 (2021). 32886587 10.1146/annurev-psych-062520-122331

[5] Cohen, S., Murphy, M. L. M. & Prather, A. A. Ten surprising facts about stressful life events and disease risk. *Annu. Rev. Psychol.* **70**, 577–597. 10.1146/annurev-psych-010418-102857 (2019). 29949726 10.1146/annurev-psych-010418-102857 PMC6996482

[6] Reif, J. A. M., Spieß, E. & Pfaffinger, K. F. in *Dealing with Stress in a Modern Work Environment: Resources Matter*. 1–18 (eds Reif, A. M., Spieß, E & Pfaffinger, K. F.) (Springer International Publishing, 2021). .

[7] Hassard, J., Teoh, K. R. H., Visockaite, G., Dewe, P. & Cox, T. The cost of work-related stress to society: A systematic review. *J. Occup. Health Psychol.* **23**, 1–17. 10.1037/ocp0000069 (2018). 28358567 10.1037/ocp0000069

[8] Lazarus, R. S. & Folkman, S. *Stress, Appraisal, and Coping* (Springer Publishing Company, 1984).

[9] Meichenbaum, D. in *Principles and Practice of Stress Management*. 3rd edn, 497–516 (eds Lehrer, P. M., Woolfolk, P. M., Paul, M. & Sime) (The Guilford Press, 2007).

[10] Jacobson, E. & Wirth, K. *Entspannung als Therapie: Progressive Relaxation in Theorie und Praxis. Aus dem Amerikanischen von Karin Wirth. Mit einem Vorwort und Nachwort von Norbert Klinkenberg*. (Klett-Cotta, 2011).

[11] Naik, G. S., Gaur, G. S. & Pal, G. K. Effect of modified slow breathing exercise on perceived stress and basal cardiovascular parameters. *Int. J. Yoga* **11**, 53–58. 10.4103/ijoy.IJOY_41_16 (2018). 10.4103/ijoy.IJOY_41_16 PMC5769199 29343931

[12] Goessl, V. C., Curtiss, J. E. & Hofmann, S. G. The effect of heart rate variability biofeedback training on stress and anxiety: A meta-analysis. *Psychol. Med.* **47**, 2578–2586. 10.1017/S0033291717001003 (2017). 28478782 10.1017/S0033291717001003

[13] Schaefer, M., Egloff, B., Gerlach, A. L. & Witthöft, M. Improving heartbeat perception in patients with medically unexplained symptoms reduces symptom distress. *Biol. Psychol.* **101**, 69–76. 10.1016/j.biopsycho.2014.05.012 (2014). 25038304 10.1016/j.biopsycho.2014.05.012

[14] Braet, J. & Braet, C. I can feel my heartbeat: The relationship between interoceptive abilities and emotional States during stress and recovery in healthy adolescents. *Psychophysiology* **61**, e14679. 10.1111/psyp.14679 (2024). 39268617 10.1111/psyp.14679

[15] Price, C. J. & Hooven, C. Interoceptive awareness skills for emotion regulation: Theory and approach of mindful awareness in Body-Oriented therapy (MABT). *Front. Psychol.*10.3389/fpsyg.2018.00798 (2018). 29892247 10.3389/fpsyg.2018.00798 PMC5985305

[16] Pinna, T. & Edwards, D. J. A systematic review of associations between interoception, vagal tone, and emotional regulation: Potential applications for mental health, wellbeing, psychological flexibility, and chronic conditions. *Front. Psychol.*10.3389/fpsyg.2020.01792 (2020). 32849058 10.3389/fpsyg.2020.01792 PMC7419655

[17] Kröll, C., Doebler, P. & Nüesch, S. Meta-analytic evidence of the effectiveness of stress management at work. *Eur. J. Work Organization. Psychol.* **26**, 677–693. 10.1080/1359432X.2017.1347157 (2017).

[18] Amanvermez, Y. et al. Stress management interventions for college students: A systematic review and meta-analysis. *Clin. Psychol. Sci. Pract.*10.1111/cpsp.12342 (2020).

[19] Alegría, M., Nakash, O. & NeMoyer, A. Increasing equity in access to mental health care: A critical first step in improving service quality. *World Psychiatry*. **17**, 43–44. 10.1002/wps.20486 (2018). 29352534 10.1002/wps.20486 PMC5775117

[20] Barbato, A., Vallarino, M., Rapisarda, F., Lora, A. & de Almeida, J. M. C. EU compass for action on mental health and well-being. *Access to mental health care in Europe. Scientific paper. Funded by the European Union in the frame of the 3rd EU Health Programme 2014-2020* (2016)

[21] Linardon, J., Firth, J., Torous, J., Messer, M. & Fuller-Tyszkiewicz, M. Efficacy of mental health smartphone apps on stress levels: A meta-analysis of randomised controlled trials. *Health Psychol. Rev.* **18**, 839–852. 10.1080/17437199.2024.2379784 (2024). 39041586 10.1080/17437199.2024.2379784

[22] Paganini, S. et al. Stress management apps: Systematic search and multidimensional assessment of quality and characteristics. *JMIR Mhealth Uhealth*. **11**, e42415. 10.2196/42415 (2023). 37642999 10.2196/42415 PMC10498318

[23] Milne-Ives, M., Selby, E., Inkster, B., Lam, C. & Meinert, E. Artificial intelligence and machine learning in mobile apps for mental health: A scoping review. *PLOS Digit. Health*. **1**, e0000079. 10.1371/journal.pdig.0000079 (2022). 36812623 10.1371/journal.pdig.0000079 PMC9931284

[24] Rafiqi, S., Wangwiwattana, C., Fernandez, E., Nair, S. & Larson, E. Work-in-Progress, PupilWare-M: Cognitive Load Estimation Using Unmodified Smartphone Cameras. In *IEEE 12th International Conference on Mobile Ad Hoc and Sensor Systems.* 645–650. 10.1109/MASS.2015.31 (Dallas, TX, USA, 2015).

[25] Cho, Y., Julier, S. J. & Bianchi-Berthouze, N. Instant stress: Detection of perceived mental stress through smartphone photoplethysmography and thermal imaging. *JMIR Mental Health*. **6**, e10140. 10.2196/10140 (2019). 30964440 10.2196/10140 PMC6477570

[26] Cho, Y., Bianchi-Berthouze, N., Julier, S. J. & Marquardt, N. ThermSense: Smartphone-based breathing sensing platform using noncontact low-cost thermal camera, 2017. In *Seventh International Conference on Affective Computing and Intelligent Interaction Workshops and Demos (ACIIW).* 83–84. 10.1109/ACIIW.2017.8272593 (San Antonio, TX, USA, 2017).

[27] Hilty, D. M., Armstrong, C. M., Luxton, D. D., Gentry, M. T. & Krupinski, E. A. A scoping review of sensors, wearables, and remote monitoring for behavioral health: Uses, outcomes, clinical competencies, and research directions. *J. Technol. Behav. Sci.* **6**, 278–313. 10.1007/s41347-021-00199-2 (2021). 10.1007/s41347-020-00190-3 PMC7819828 33501372

[28] Allen, A. P., Kennedy, P. J., Cryan, J. F., Dinan, T. G. & Clarke, G. Biological and psychological markers of stress in humans: Focus on the Trier social stress test. *Neurosci. Biobehav. Rev.* **38**, 94–124. 10.1016/j.neubiorev.2013.11.005 (2014). 24239854 10.1016/j.neubiorev.2013.11.005

[29] Yu, B., Funk, M., Hu, J., Wang, Q. & Feijs, L. Biofeedback for everyday stress management: A systematic review. *Front. ICT*10.3389/fict.2018.00023 (2018).

[30] Nelson, B. W. et al. Smartphone photoplethysmography pulse rate covaries with stress and anxiety during a digital acute social stressor. *Psychosom. Med.*10.1097/PSY.0000000000001178 (2023). 37409791 10.1097/PSY.0000000000001178

[31] Schultchen, D., Bayer, J., Kühnel, J., Melchers, K. G. & Pollatos, O. Interoceptive accuracy is related to long-term stress via self-regulation. *Psychophysiology* **56**, e13429. 10.1111/psyp.13429 (2019). 31231829 10.1111/psyp.13429

[32] Economides, M. et al. Feasibility and efficacy of the addition of heart rate variability biofeedback to a remote digital health intervention for depression. *Appl. Psychophysiol. Biofeedback*. **45**, 75–86. 10.1007/s10484-020-09458-z (2020). 32246229 10.1007/s10484-020-09458-z PMC7250954

[33] Latour, C. et al. Improving mental health in U.S. Veterans using mHealth tools: A pilot study. *Health Inf. J.*10.1177/1460458220954613 (2020). 10.1177/1460458220954613 PMC8112186 32972313

[34] Ponzo, S. et al. Efficacy of the digital therapeutic mobile app biobase to reduce stress and improve mental Well-Being among university students: Randomized controlled trial. *JMIR Mhealth Uhealth*. **8**, e17767. 10.2196/17767 (2020). 31926063 10.2196/17767 PMC7171562

[35] Baig, M. M., GholamHosseini, H., Moqeem, A. A., Mirza, F. & Lindén, M. A. Systematic review of wearable patient monitoring Systems – Current challenges and opportunities for clinical adoption. *J. Med. Syst.* **41**, 115. 10.1007/s10916-017-0760-1 (2017). 28631139 10.1007/s10916-017-0760-1

[36] Lin, B., Prickett, C. & Woltering, S. Feasibility of using a biofeedback device in mindfulness training - a pilot randomized controlled trial. *Pilot Feasib. Stud.* **7**, 84. 10.1186/s40814-021-00807-1 (2021). 10.1186/s40814-021-00807-1 PMC7988913 33762016

[37] Kranjec, J., Beguš, S., Geršak, G. & Drnovšek, J. Non-contact heart rate and heart rate variability measurements: A review. *Biomed. Signal Process. Control*. **13**, 102–112. 10.1016/j.bspc.2014.03.004 (2014).

[38] Li, K. H. C. et al. The current state of mobile phone apps for monitoring heart rate, heart rate variability, and atrial fibrillation: Narrative review. *JMIR Mhealth Uhealth*. **7**, e11606. 10.2196/11606 (2019). 30767904 10.2196/11606 PMC6396075

[39] Yoon, S. I., Lee, S. I., Suh, H. W., Chung, S. Y. & Kim, J. W. Effects of mobile mindfulness training on mental health of employees: A CONSORT-compliant pilot randomized controlled trial. *Medicine* **101**, e30260. 10.1097/md.0000000000030260 (2022). 36107583 10.1097/MD.0000000000030260 PMC9439769

[40] Maeda, Y., Sekine, M. & Tamura, T. Relationship between measurement site and motion artifacts in wearable reflected photoplethysmography. *J. Med. Syst.* **35**, 969–976. 10.1007/s10916-010-9505-0 (2011). 20703691 10.1007/s10916-010-9505-0

[41] Pai, A., Veeraraghavan, A. & Sabharwal, A. HRVCam: Robust camera-based measurement of heart rate variability. *J. Biomed. Opt.* **26**, 022707–022707. 10.1117/1.JBO.26.2.022707 (2021). 33569935 10.1117/1.JBO.26.2.022707 PMC7874852

[42] Cho, D., Kim, J., Lee, K. J. & Kim, S. Reduction of motion artifacts from remote photoplethysmography using adaptive noise cancellation and modified HSI model. *IEEE Access.* **9**, 122655–122667. 10.1109/ACCESS.2021.3106046 (2021).

[43] Şengül, G., Ozcelik, E., Misra, S., Damaševičius, R. & Maskeliūnas, R. Fusion of smartphone sensor data for classification of daily user activities. *Multimedia Tools Appl.*10.1007/s11042-021-11105-6 (2021).

[44] Eysenbach, G. CONSORT-EHEALTH: Improving and standardizing evaluation reports of Web-based and mobile health interventions. *J. Med. Internet Res.* **13**, e126. 10.2196/jmir.1923 (2011). 22209829 10.2196/jmir.1923 PMC3278112

[45] Faul, F., Erdfelder, E., Buchner, A. & Lang, A. G. Statistical power analyses using G* power 3.1: Tests for correlation and regression analyses. *Behav. Res. Methods*. **41**, 1149–1160. 10.3758/BRM.41.4.1149 (2009). 19897823 10.3758/BRM.41.4.1149

[46] Heber, E. et al. The benefit of web-and computer-based interventions for stress: A systematic review and meta-analysis. *J. Med. Internet. Res.* **19**, e32. 10.2196/jmir.5774 (2017). 28213341 10.2196/jmir.5774 PMC5336602

[47] Ly, K. H., Asplund, K. & Andersson, G. Stress management for middle managers via an acceptance and commitment-based smartphone application: A randomized controlled trial. *Internet Intervent.*. **1**, 95–101. 10.1016/j.invent.2014.06.003 (2014).

[48] Romas, J. A. & Sharma, M. in *Practical Stress Management (Seventh Edition)* (eds Romas, J. A. & Sharma, M.) 169–188 (Academic Press, 2017).

[49] Nezu, A. M., Nezu, C. M. & D’Zurilla, T. *Problem-Solving Therapy: A Treatment Manual* (Springer Publishing Company, 2012).

[50] Berking, M. & Whitley, B. *Affect Regulation Training: A Practitioners’ Manual* (Springer, 2016).

[51] Heinrichs, M., Stächele, T. & Domes, G. *Stress und Stressbewältigung* (Hogrefe Verlag, 2015).

[52] Kabat-Zinn, J. Mindfulness-based interventions in context: Past, present, and future. *Clin. Psychol. Sci. Pract.* **10**, 144–156. 10.1093/clipsy.bpg016 (2003).

[53] Specht, M. B., Spaude, E. & Kaluza, A. *Kurzintervention bei Insomnie (KI): Eine Anleitung zur Behandlung von Ein- und Durchschlafstörungen* (Kohlhammer Verlag, 2014).

[54] Wagner-Link, A. *Verhaltenstraining zur Stressbewältigung: Arbeitsbuch für Therapeuten und Trainer; [inklusive CD mit Arbeitsblättern]* (Klett-Cotta, 2010).

[55] Heber, E., Lehr, D., Ebert, D. D., Berking, M. & Riper, H. Web-Based and mobile stress management intervention for employees: A randomized controlled trial. *J. Med. Internet Res.* **18**, e21. 10.2196/jmir.5112 (2016). 26818683 10.2196/jmir.5112 PMC4749847

[56] Ebert, D. D. et al. Internet- and mobile-based stress management for employees with adherence-focused guidance: Efficacy and mechanism of change. *Scand J Work Environ Health*10.5271/sjweh.3573 (2016). 27249161 10.5271/sjweh.3573

[57] Ebert, D. D. et al. Self-guided internet-based and mobile-based stress management for employees: Results of a randomised controlled trial. *Occup. Environ. Med.* **73**, 315–323. 10.1136/oemed-2015-103269 (2016). 26884049 10.1136/oemed-2015-103269

[58] Carey, T. A. & Mullan, R. J. What is socratic questioning? *Psychother. Theory Res. Pract. Train.* **41**, 217–226. 10.1037/0033-3204.41.3.217 (2004).

[59] Loijen, A., Vrijsen, J. N., Egger, J. I. M., Becker, E. S. & Rinck, M. Biased approach-avoidance tendencies in psychopathology: A systematic review of their assessment and modification. *Clin. Psychol. Rev.* **77**, 101825. 10.1016/j.cpr.2020.101825 (2020). 32143108 10.1016/j.cpr.2020.101825

[60] Ruf, T., Ernst, A. & Küblbeck, C. in *Microelectronic Systems: Circuits, Systems and Applications* 243–252 (eds Heuberger, A., Elst, G. & Hanke, R.) (Springer, 2011).

[61] Jeong, J. G., Kang, S. W. & Choi, S. B. Employees’ weekend activities and psychological Well-Being via job stress: A moderated mediation role of recovery experience. *Int. J. Environ. Res. Public Health*. **17**, 1642 (2020). 32138361 10.3390/ijerph17051642 PMC7084709

[62] Boucher, E. M. & Raiker, J. S. Engagement and retention in digital mental health interventions: A narrative review. *BMC Digit. Health*. **2**, 52. 10.1186/s44247-024-00105-9 (2024).

[63] Chu, B., Marwaha, K., Sanvictores, T., Awosika, A. O. & Ayers, D. *StatPearls* (StatPearls Publishing, 2024). 31082164

[64] Pinheiro, E., Postolache, O. & Girão, P. Theory and developments in an unobtrusive cardiovascular system representation: Ballistocardiography. *Open. Biomed. Eng. J.* **4**, 201–216. 10.2174/1874120701004010201 (2010). 21673836 10.2174/1874120701004010201 PMC3111731

[65] Landreani, F. & Caiani, E. G. Smartphone accelerometers for the detection of heart rate. *Expert Rev. Med. Devices*. **14**, 935–948. 10.1080/17434440.2017.1407647 (2017). 29161916 10.1080/17434440.2017.1407647

[66] Hernandez, J., McDuff, D., Quigley, K., Maes, P. & Picard, R. W. Wearable motion-based heart rate at rest: A workplace evaluation. *IEEE J. Biomed. Health Inf.* **23**, 1920–1927. 10.1109/JBHI.2018.2877484 (2019). 10.1109/JBHI.2018.2877484 30387751

[67] Hernandez, J., McDuff, D. J. & Picard, R. W. Biophone: Physiology monitoring from peripheral smartphone motions. In *37th Annual International Conference of the IEEE Engineering in Medicine and Biology Society (EMBC)* 7180–7183. 10.1109/EMBC.2015.7320048 (Milan, Italy, 2015). 10.1109/EMBC.2015.7320048 26737948

[68] Rummel, B. *System Usability Scale – jetzt auch auf Deutsch.* (2016). https://community.sap.com/t5/additional-blogs-by-sap/system-usability-scale-jetzt-auch-auf-deutsch/ba-p/13487686

[69] Klein, E. M. et al. The German version of the perceived stress Scale–psychometric characteristics in a representative German community sample. *BMC Psychiatry*. **16**, 159. 10.1186/s12888-016-0875-9 (2016). 27216151 10.1186/s12888-016-0875-9 PMC4877813

[70] Berking, M. & Znoj, H. Entwicklung und validierung eines Fragebogens Zur standardisierten Selbsteinschätzung emotionaler kompetenzen (SEK-27). *Z. Für Psychiatrie Psychologie Und Psychother.* **56**, 141–153. 10.1024/1661-4747.56.2.141 (2008).

[71] Martin, A., Rief, W., Klaiberg, A. & Braehler, E. Validity of the brief patient health questionnaire mood scale (PHQ-9) in the general population. *Gen. Hosp. Psychiatry*. **28**, 71–77. 10.1016/j.genhosppsych.2005.07.003 (2006). 16377369 10.1016/j.genhosppsych.2005.07.003

[72] Brähler, E., Mühlan, H., Albani, C. & Schmidt, S. Teststatistische Prüfung und normierung der Deutschen versionen des EUROHIS-QOL lebensqualität-Index und des WHO-5 wohlbefindens-index. *Diagnostica* **53**, 83–96. 10.1026/0012-1924.53.2.83 (2007).

[73] Bates, D., Mächler, M., Bolker, B. & Walker, S. Fitting linear mixed-effects models using lme4. *arXiv preprint *arXiv:1406.5823 (2015).

[74] Raudenbush, S. W. *Hierarchical Linear Models: Applications and Data Analysis Methods* Vol. 1 (sage, 2002).

[75] Graham, J. W. Missing data analysis: Making it work in the real world. *Ann. Rev. Psychol.* **60**, 549–576. 10.1146/annurev.psych.58.110405.085530 (2009). https://doi.org/https://doi.org/18652544 10.1146/annurev.psych.58.110405.085530

[76] Kuznetsova, A., Brockhoff, P. B. & Christensen, R. H. B. LmerTest package: Tests in linear mixed effects models. *J. Stat. Softw.* **82**, 1–26. 10.18637/jss.v082.i13 (2017).

[77] Feingold, A. Effect sizes for growth-modeling analysis for controlled clinical trials in the same metric as for classical analysis. *Psychol. Methods*. **14**, 43–53. 10.1037/a0014699 (2009). 19271847 10.1037/a0014699 PMC2712654

[78] Benjamini, Y. & Hochberg, Y. Controlling the false discovery rate: A practical and powerful approach to multiple testing. *J. Roy. Stat. Soc.: Ser. B (Methodol.)*. **57**, 289–300. 10.1111/j.2517-6161.1995.tb02031.x (1995).

[79] Bangor, A., Kortum, P. T. & Miller, J. T. An empirical evaluation of the system usability scale. *Int. J. Human–Computer Interact.* **24**, 574–594. 10.1080/10447310802205776 (2008).

[80] Linardon, J., Cuijpers, P., Carlbring, P., Messer, M. & Fuller-Tyszkiewicz, M. The efficacy of app‐supported smartphone interventions for mental health problems: A meta‐analysis of randomized controlled trials. *World Psychiatry*. **18**, 325–336. 10.1002/wps.20673 (2019). 31496095 10.1002/wps.20673 PMC6732686

[81] Zaccaro, A. et al. How Breath-Control can change your life: A systematic review on Psycho-Physiological correlates of slow breathing. *Front. Hum. Neurosci.*10.3389/fnhum.2018.00353 (2018). 30245619 10.3389/fnhum.2018.00353 PMC6137615

[82] Schumann, A., de la Cruz, F., Köhler, S., Brotte, L. & Bär, K. J. The influence of heart rate variability biofeedback on cardiac regulation and functional brain connectivity. *Front. NeuroSci.* **15**10.3389/fnins.2021.691988 (2021). 34267625 10.3389/fnins.2021.691988 PMC8275647

[83] Kennedy, L. & Parker, S. H. Biofeedback as a stress management tool: A systematic review. *Cogn. Technol. Work*. **21**, 161–190. 10.1007/s10111-018-0487-x (2019).

[84] ter Harmsel, J. F. et al. Biocueing and ambulatory biofeedback to enhance emotion regulation: A review of studies investigating non-psychiatric and psychiatric populations. *Int. J. Psychophysiol.* **159**, 94–106. 10.1016/j.ijpsycho.2020.11.009 (2021). 33248196 10.1016/j.ijpsycho.2020.11.009

[85] Perna, G. et al. Heart rate variability: Can it serve as a marker of mental health resilience? Special section on translational and neuroscience studies in affective disorders section editor, Maria nobile MD, phd. *J. Affect. Disord.* **263**, 754–761. 10.1016/j.jad.2019.10.017 (2020). 31630828 10.1016/j.jad.2019.10.017

[86] Kim, J. W., Seok, H. S. & Shin, H. Is ultra-short-term heart rate variability valid in non-static conditions?. *Front. Physiol.*10.3389/fphys.2021.596060 (2021). 33859568 10.3389/fphys.2021.596060 PMC8042416

[87] Linardon, J. & Fuller-Tyszkiewicz, M. Attrition and adherence in smartphone-delivered interventions for mental health problems: A systematic and meta-analytic review. *J. Consult Clin. Psychol.* **88**, 1–13. 10.1037/ccp0000459 (2020). 31697093 10.1037/ccp0000459

[88] Linardon, J. Rates of attrition and engagement in randomized controlled trials of mindfulness apps: Systematic review and meta-analysis. *Behav. Res. Ther.* **170**, 104421. 10.1016/j.brat.2023.104421 (2023). 37862854 10.1016/j.brat.2023.104421

[89] Torous, J., Nicholas, J., Larsen, M. E., Firth, J. & Christensen, H. Clinical review of user engagement with mental health smartphone apps: Evidence, theory and improvements. *Evid. Based Mental Health*. **21**, 116–119. 10.1136/eb-2018-102891 (2018). 10.1136/eb-2018-102891 PMC10270395 29871870

[90] Constantino, M. J., Vîslă, A., Coyne, A. E. & Boswell, J. F. A meta-analysis of the association between patients’ early treatment outcome expectation and their posttreatment outcomes. *Psychotherapy* **55**, 473 (2018). 30335459 10.1037/pst0000169

[91] Wampold, B. E. How important are the common factors in psychotherapy? An update. *World Psychiatry*. **14**, 270–277. 10.1002/wps.20238 (2015). 26407772 10.1002/wps.20238 PMC4592639

[92] Stalujanis, E. et al. Induction of efficacy expectancies in an ambulatory Smartphone-Based digital placebo mental health intervention: Randomized controlled trial. *JMIR Mhealth Uhealth*. **9**, e20329. 10.2196/20329 (2021). 33594991 10.2196/20329 PMC7929742

[93] Stone, A. A., Bachrach, C. A., Jobe, J. B., Kurtzman, H. S. & Cain, V. S. *The science of self-report: Implications for research and practice* (Psychology Press, UK, 1999).

[94] Haque, Y. et al. State-of-the-Art of stress prediction from heart rate variability using artificial intelligence. *Cogn. Comput.* **16**, 455–481. 10.1007/s12559-023-10200-0 (2024).

[95] Weisel, K. K. et al. Standalone smartphone apps for mental health—a systematic review and meta-analysis. *Npj Digit. Med.* **2**, 118. 10.1038/s41746-019-0188-8 (2019). 31815193 10.1038/s41746-019-0188-8 PMC6889400

[96] Fuhrmann, L. M. et al. Additive effects of adjunctive app-based interventions for mental disorders - A systematic review and meta-analysis of randomised controlled trials. *Internet Intervent.* **35**, 100703. 10.1016/j.invent.2023.100703 (2024). 10.1016/j.invent.2023.100703 PMC10788289 38225971

[97] Lau, N. et al. Android and iPhone mobile apps for psychosocial wellness and stress management: Systematic search in app stores and literature review. *JMIR Mhealth Uhealth*. **8**, e17798. 10.2196/17798 (2020). 32357125 10.2196/17798 PMC7275252

