# Supplementary material for: Evaluating a brief smartphone-based stress management intervention with heart rate biofeedback from built-in sensors in a three arm randomized controlled trial
Source: Sci Rep. 2025 Jun 23;15:20257. doi: 10.1038/s41598-025-06588-4 (PMC12185712; doi:10.1038/s41598-025-06588-4)
Supplement: Supplementary file 1 — Supplementary Material 1 [file 41598_2025_6588_MOESM1_ESM.pdf]

Supplemental Materials

Evaluating a brief smartphone based stress management intervention with heart rate biofeedback from built in sensors in a three arm randomized controlled trial

By Lukas M. Fuhrmann[1]. Christian Aljoscha Lukas[2]. Lena Schindler-Gmelch[1]. and Matthias Berking[1]

[1]Department of Clinical Psychology and Psychotherapy. Friedrich-Alexander Universität Erlangen-Nürnberg. Erlangen. Germany

[2]mentalis GmbH, Nürnberg, Germany

MT-StressLess with HR-based biofeedback: Feasibiilty study relaxation exercise

Prior to the main study, we conducted a feasibility study with nine participants (*M* age = 28.5 years, SD = 12.3; n = 4 females) to assess the feasibility of the HR-based biofeedback component and derive cutoff scores for the stress and relaxation phases. For these recordings, visual inspection of Bland-Altman plots and correlational analyses demonstrated that the smartphone-based assessment of HR was strongly associated with recordings derived via conventional electrocardiography (Figure S1). HR indicators of both approaches correlated with $r = .95$ ($p < 0.001$) in a lying position and $r = 0.91$ ($p < 0.001$) in a sitting position (Table S2).

Figure S1

Bland-Altman diagrams for heart rate measurements using EKG and Ballistocardiography for four different body postures

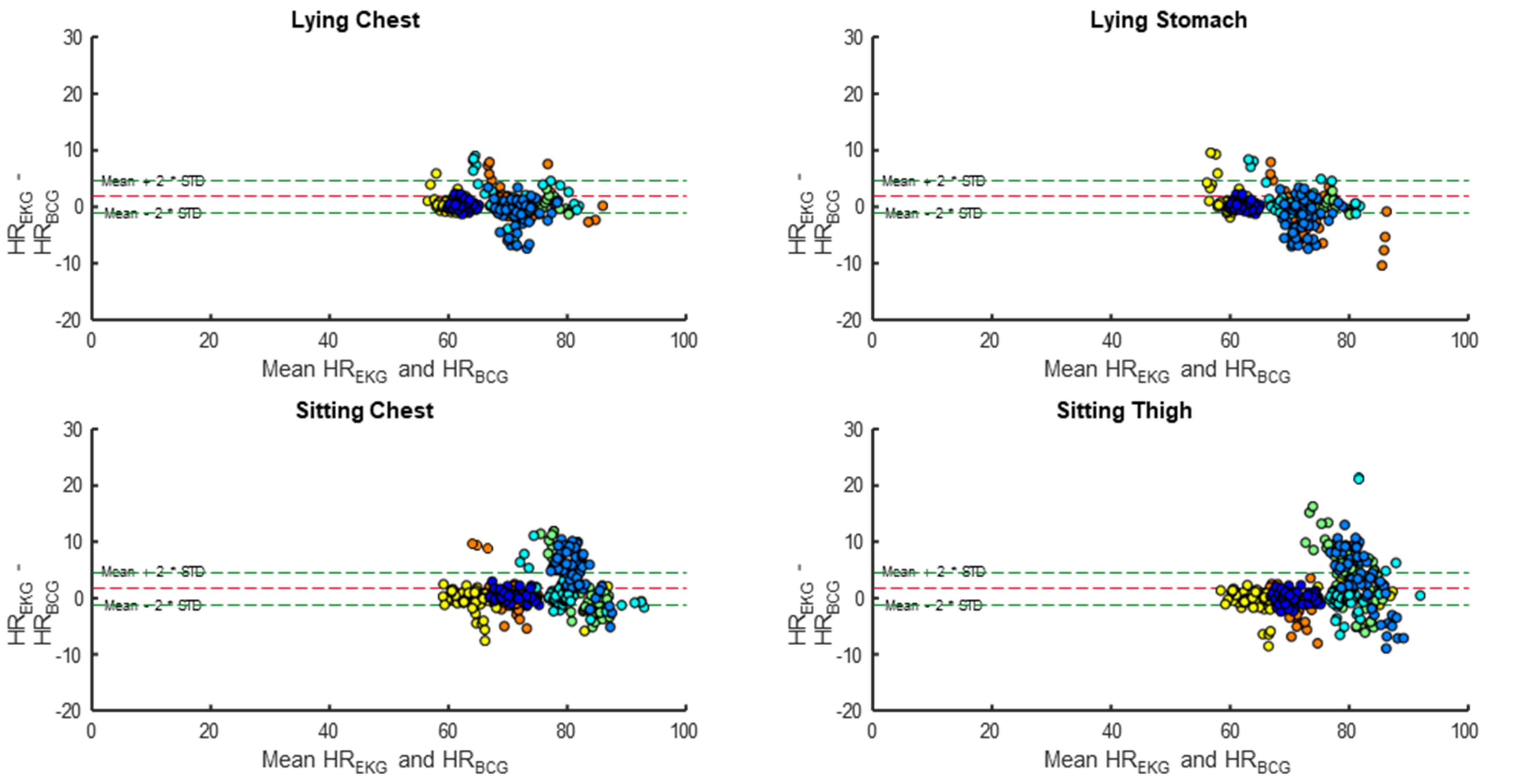

Note. BCG = ballistocardiography; EKG = electrocardiography; HR = heart rate. Each color corresponds to a unique participant-session combination, allowing visualization of potential variations between subjects and sessions.

Table S1

Accuracy of the Measurement Method of Ballistocardiography Compared to Electrocardiography in Different Body Postures

	Error in beats per minute of ballistocardiography compared to electrocardiogram		Values obtained through ballistocardiography. characterized by low quality as determined by the application programming interface (API) of GlobalVitals		Correlation of ballistocardiography with electrocardiogram all with $p < 0.001$
Body Posture	*M*	*SD*	*n*	%	*r*
Lying posture and smartphone on the chest (n measurements = 5625)	1.11	1.23	9	0.16	0.95
Lying posture and smartphone on the stomach (n = 5789)	1.30	1.43	11	0.19	0.94
Sitting posture and smartphone on the chest (n = 4130)	2.19	1.67	57	1.38	0.91
Sitting posture and smartphone on the thigh (n = 3659)	2.73	1.45	116	3.17	0.85

Table S2
Information about the steering modi in the gamified AAMT

Control option	Name	Description of user interaction control option for functional/dysfunctional stimuli
Touchscreen	Swipe	Swiping the stimuli towards/away from oneself by using finger touch
	Draw	Drawing a checkmark/crossing a stimulus out by using finger touch
	Plus-Minus	Selecting a plus sign on the bottom/a minus sign on the top of the screen by using finger touch
	Select	Selecting the functional of two simultaneously presented stimuli
Voice recognition	Command	Classifying a stimulus as good/bad; correct/wrong; future/past by saying those words out loud
Emotion recognition	Emotion recognition	Showing specific emotional facial expressions in response to a stimulus Happy/sad, angry

Note. AAMT = approach-avoidance modification training.

Table S3
Task Information and Examples

Task Modi	Example
Audio	"Relaxation often is the key to more serenity. If you want to become more calm and more relaxed. find a place where you won't be disturbed and practice the long form of progressive muscle relaxation."
Record	"Think of something that can help you deal with stress even better in the future. Say it out loud and record it as an audio file. It can be helpful to listen to your recording in the future."
Text	"Remember a moment when you had to do everything at once. Write down the thoughts and feelings that you had at that time. Make yourself aware that such a condition is not good for your health in the long run. Plan to prioritize your tasks in the future to support you on your way to a more stress-free life."
List	"A list of positive activities can serve as a collection of ideas for the future when you want to take a break from the daily grind with the help of a positive activity. List at least 20 activities. and. most importantly: Try one of these activities today."
Photo	"Take a trip into nature. Try to enjoy what you see. hear. and feel consciously. On your trip. take a picture of something beautiful that you see. Upload the picture here."
Read and act	"Think of a specific step you want to take towards your current goal - and then put it into action."

Measures

Primary Outcome

The primary outcome measure of this study was the Perceived Stress Scale-10 (PSS-10; German Version: Klein et al., 2016[1]). This self-report instrument assesses the degree of perceived stress using ten items (e.g., " In the last month, how often have you felt nervous and stressed?") to be rated on a five-point Likert scale (ranging from 0 = *never* to 4 = *very often*). The PSS-10 demonstrated acceptable-to-good internal consistency (Cronbach's α = .78[2] and α =.84[1]). In the current sample, the scale showed high internal consistency with Cronbach's α = .88 at baseline, α = .86 at postintervention, and α = .87 at follow-up.

Secondary Outcomes

We used the Emotion Regulation Skills Questionnaire (ERSQ-27; German version: Berking & Znoj, 2008[3]) to assess the successful application of adaptive emotion regulation skills (arguably an important component of coping with stress). This measure requires respondents to rate 27 items on a scale ranging from 0 (*not at all*) to 4 (*absolutely true*). Higher average scores indicate greater use of adaptive emotion regulation skills. In previous research, the ERSQ-27 has been shown to have high to excellent internal consistency (Cronbach's α = 0.94 – 0.97[4-6]). In the current sample, the scale showed excellent internal consistency with Cronbach's α = 0.92 at baseline, α = 0.94 at postintervention, and α = 0.95 at follow-up.

To assess the severity of depressive symptoms, we used the Patient Health Questionnaire (PHQ-9; German version: Martin et al., 2006[7]). The PHQ-9 requires respondents to rate nine items ranging from 0 (*not at all*) to 3 (*nearly every day*). Higher scores indicate more severe symptoms of depression. Internal consistency has been reported to be high (Cronbach's α = 0.89; Martin et al., 2006[7]). In the present sample, internal consistency was good with Cronbach's α = 0.81 at baseline, α = 0.82 at postintervention, and α = 0.82 at follow-up.

The WHO-5 Well-Being Index (WHO-5; German version: Brähler et al., 2007[8]) was used to assess subjective well-being. For this measure, respondents rate each of the five items on a scale ranging from 0 (*never*) to 5 (*all the time*), with lower values indicating poorer well-being. In previous research, the scale's internal consistency was excellent (Cronbach's α = 0.92[8]). In the present study, internal consistency was good with Cronbach's α = 0.82 at baseline, α = 0.80 at postintervention, and α = 0.87 at follow-up.

Usability

To assess usability feedback, participants in both active conditions completed the system usability scale (SUS; German version: Rummel, 2016[9]) at postintervention. The SUS uses ten items on a scale ranging from 1 (*strongly disagree*) to 5 (*strongly agree*). The usability score ranges from 0 to 100, where higher scores reflect greater perceived usability. In the present study, internal consistency was good with Cronbach's $\alpha = 0.85$ at postintervention. Furthermore, participants were asked to respond to three self-developed questions assessing comprehensibility (e.g., "The explanations in the chat format were easy to understand"), appeal (e.g., "The presentation in the chat format was appealing"), and goal achievement (e.g., "The explanations in the chat format were useful in achieving my goal (knowledge acquisition)") in relation to the specific content of the intervention, which included psychoeducation, quizzes, AAMT tasks, daily-life tasks, and the biofeedback-based relaxation exercise. Responses were rated on a scale ranging from 1 (*strongly disagree*) to 5 (*strongly agree*)

Table S4

Sociodemographic and Clinical Characteristics of Completers and None-Completers at Postintervention

	Completers (n = 130)		None-completers (n = 36)		Statistics			
Variable					*df*	$x^2/F/t$	*p*	R^2/V
Gender					2	2.35	0.31	0.12
Female (*n,* %)	104	80	25	69.44				
Male (*n,* %)	25	19.23	11	30.56				
Diverse (*n,* %)	1	0.77	0	0				
Age (*M. SD*)	23.3	7.21	27.17	9.84	45.89	2.2	0.033	
Age (range)	18-60		18-59					
Highest education degree					5	5.97	0.31	0.19
None (*n,* %)	1	0.77	0	0				
Secondary General School (*n,* %)	2	1.54	0	0				
Intermediate Secondary School (*n,* %)	3	2.31	3	8.33				
Graduate (*n,* %)	90	69.23	21	58.33				
Bachelor or Master Degree (*n,* %)	31	23.85	12	33.33				
PhD (*n,* %)	3	2.31	0	0				
Employement					3	0.77	0.86	0.07
Employed (*n,* %)	9	6.92	4	11.11				
Unemployed (*n,* %)	3	2.31	1	2.78				
Student (*n,* %)	93	71.54	25	69.44				
Others (*n,* %)	25	19.23	6	16.67				
Health-related variables								
Current psychological or psychiatric treatment (*n,* %)	10	7.69	3	8.33	Fisher's exact test		1	
Current cardiovascular disease (*n,* %)	6	4.62	1	2.78	Fisher's exact test		1	
Currently smoking cessation (*n,* %)	11	8.46	1	2.78	Fisher's exact test		0.74	
Physical activity per week, in hours (*M, SD*)	3.64	3.5	3.56	3.6	50.39	0.12	0.90	
Experience in relaxation exercises (*n,* %)	80	61.54	21	58.33	Fisher's exact test		0.85	
Frequency of smartphone use in daily life (*M, SD*)	4.18	0.8	4.22	0.8	58.33	0.3	0.77	
PSS-10 at screening (*M, SD*)	22.17	6.7	22.5	7.3	52.35	0.25	0.81	

Note. Frequency of smartphone use was assed via the self-developed question “How often do you use your smartphone?”; item range: 1 (very rarely) – 5 (very often); PSS-10 = Perceived Stress Scale.

Table S5

Sociodemographic and Clinical Characteristics of Completers and None-Completers at Follow-up

	Completer (n = 128)		None-completer (n = 38)		Statistics			
Variable					*df*	*x²/F/t*	*p*	*R²/V*
Gender					2	3.07	0.22	0.14
Female (*n.* %)	103	80.47	26	68.42				
Male (*n.* %)	24	18.75	12	31.58				
Diverse (*n.* %)	1	0.78	0	0				
Age (*M, SD*)	23.34	7.26	26.84	9.68	49.98	2.07	0.04	
Age (range)	18-60		18-59					
Highest education degree					5	5.19	0.38	0.18
None (*n,* %)	1	0.78	0	0				
Secondary General School (*n,* %)	2	1.56	0	0				
Intermediate Secondary School (*n,* %)	3	2.34	3	7.89				
Graduate (*n,* %)	88	68.75	23	60.53				
Bachelor or Master Degree (*n,* %)	31	24.22	12	31.58				
PhD (*n,* %)	3	2.34	0	0				
Employement					3	0.69	0.88	0.64
Employed (*n,* %)	9	7.03	4	10.53				
Unemployed (*n,* %)	3	2.34	1	2.63				
Student (*n,* %)	91	71.09	27	71.05				
Others (*n,* %)	25	19.53	6	15.79				
Health-related variables								
Current psychological or psychiatric treatment (*n,* %)	10		3		Fisher's exact test		1	
Current cardiovascular disease (*n,* %)	1		6		Fisher's exact test		1	
Currently smoking cessation (*n,* %)	11		2		Fisher's exact test		0.73	
Physical activity per week, in hours (*M, SD*)	3.6	3.46	3.69	3.55	55.73	0.13	0.89	
Experience in relaxation exercises (*n,* %)	80		21		Fisher's exact test		0.45	
Frequency of smartphone use in daily life (*M, SD*)	4.17	0.84	4.24	0.79	64.37	0.44	0.66	
PSS-10 at screening (*M, SD*)	22.29	6.6	22.08	7.6	54.53	-0.16	0.88	

Note. Frequency of smartphone use was assed via the self-developed question "How often do you use your smartphone?"; item range: 1 (very rarely) – 5 (very often); PSS-10 = Perceived Stress Scale.

Table S6

Usage of App Features. Differences Between the Active Conditions and Correlation with Outcome

	MT-StressLess + biofeedback (n = 49)		*MT-StressLess* (n = 47)		Both Apps (n = 96)		x^2/Wilcoxon rank sum test (*MT-StressLess* + biofeedback vs. *MT-StressLess*)		Correlation with Post-intervention PSS-10 values			
									MT-StressLess + biofeedback		*MT-StressLess*	
	M/*n*	*SD*/%	*M*/*n*	*SD*/%	*M*/*n*	*SD*/%	x^2/*W*	*p*	r_s	*p*	r_s	*p*
Completed 14 competencies (*n, %*)	11	22.45	14	29.79	25	26.04	0.32	0.572	-	-	-	-
Completed at least 7 competencies (*n, %*)	24	48.98	27	57.45	51	53.12	0.16	0.692	-	-	-	-
Completed competencies (*M, SD*)	6.80	5.51	8.13	4.97	7.45	5.27	949	0.134	-0.25	.195	-0.29	0.082
Active usage days (*M, SD*)	10.80	5.60	12.60	4.82	11.70	5.29	961	0.162	-0.14	.428	-0.36	0.038
Minutes spent in the app (*M, SD*)	150	129	119	65.50	135	104	1192.5	0.767	-0.29	.062	**-0.41**	0.025
Completed psychoeducation quizzes (*M, SD*)	15.10	12	16.60	9.44	15.80	10.80	1008.5	0.296	-0.13	.428	-0.33	0.059
Solved AAMT games (*M, SD*)	8.20	6.21	9.47	5.13	8.82	5.71	990	0.236	-0.30	.139	**-0.36**	0.038
Solved tasks (*M, SD*)	60.40	67.30	70.90	61.10	65.50	64.20	916.5	0.086	-0.19	.340	-0.16	0.349
HR biofeedback exercise completed (*M, SD*)	5.22	3.82	-	-	-	-	-	-	-0.35	.073	-	-

Note. All data in this table are non-normally distributed. Wilcoxon rank-sum tests were used to detect differences between the active conditions. Spearman's rank correlations was used to detect correlation between app usage and postintervention outcomes, which were residualized by their preintervention values. The Benjamini-Hochberg correction was applied to adjust the p-values for multiple comparisons, controlling the false discovery rate. AAMT = approach-avoidance modification training; MT-StressLess + biofeedback = Mentalis StressLess app-based intervention condition with heart rate-based biofeedback; MT-StressLess = Mentalis StressLess app-based intervention condition without heart rate-based biofeedback.

Table S7

Results for Items on Usability and Comprehensibility

Item	N	Minimum	Maximum	Mean	Std. Deviation
The explanations in the chat format were easy to understand.	82	2	5	4.84	0.46
The quiz questions in the chat format were easy to understand.	82	1	5	4.82	0.61
The stimuli in the brain games were easy to understand.	82	2	5	4.57	0.72
The audio instructions were easy to understand.	82	1	5	4.74	0.66
The brain game "Swipe" was easy to understand.	82	1	5	4.72	0.71
The brain game "Language" was easy to understand.	59	1	5	4.32	1.07
The brain game "Drawing" was easy to understand.	59	3	5	4.85	0.45
The brain game "Select" was easy to understand.	36	2	5	4.81	0.62
The brain game "Plus & Minus" was easy to understand.	64	3	5	4.55	0.71
The brain game "Emotion Recognition" was easy to understand.	64	1	5	4.28	1.03
The tasks were easy to understand.	82	2	5	4.71	0.59
The practice instructions for the relaxation exercise with heart rate measurement were easy to understand.	40	1	5	4.28	1.04

Table S8

Results for Items on Usability and Appeal

Item	N	Minimum	Maximum	Mean	Std. Deviation
The presentation in the chat format was appealing.	82	1	5	3.80	1.14
The quiz questions in the chat flow were appealing.	82	1	5	3.21	1.24
The stimuli (images and sentences) in the brain games were appealing.	82	1	5	4.17	0.90
The audio instructions were appealing.	82	1	5	3.94	1.07
The brain game "Swipe" (swiping stimuli away or pulling them closer) was appealing.	82	2	5	4.22	0.98
The brain game "Language" (voice control) was appealing.	59	1	5	2.73	1.27
The brain game "Drawing" (crossing out or checkmarks) was appealing.	59	2	5	4.56	0.70
The brain game "Select" (tapping the positive stimulus) was appealing.	36	3	5	4.53	0.61
The brain game "Plus & Minus" (tapping + or -) was appealing.	64	1	5	4.06	0.89
The brain game "Emotion Recognition" (control via facial emotion recognition) was appealing.	64	1	5	2.73	1.45
The presentation of the tasks was appealing.	82	1	5	4.24	0.98
The relaxation exercise with heart rate measurement was appealing.	40	1	5	3.35	1.41

Table S9

Results for Items on Usability and Goal Achievement

Item	N	Minimum	Maximum	Mean	Std. Deviation
The explanations in the chat format were useful for achieving my goal (knowledge acquisition).	82	1	5	3.90	1.04
The quiz questions in the chat were useful for achieving my goal (knowledge acquisition).	82	1	5	3.54	1.20
The stimuli in the brain games were useful for achieving my goal.	82	1	5	3.99	1
The audio instructions were useful for achieving my goal.	82	1	5	4.11	0.96
The brain game "Swipe" was useful for achieving my goal.	82	1	5	3.76	1.06
The brain game "Language" was useful for achieving my goal.	59	1	5	3.08	1.24
The brain game "Drawing" was useful for achieving my goal.	59	1	5	4.12	0.89
The brain game "Select" was useful for achieving my goal.	36	3	5	4.28	0.66
The brain game "Plus & Minus" was useful for achieving my goal.	64	2	5	3.88	0.88
The brain game "Emotion Recognition" was useful for achieving my goal.	64	1	5	3.11	1.31
The tasks were useful for achieving my goal.	82	1	5	3.89	1.01
The relaxation exercise with heart rate measurement was useful for achieving my goal.	40	1	5	2.85	1.19

Table S10

Observed Means for Primary and Secondary Outcome Measures

Outcome	*MT-StressLess* + biofeedback			*MT-StressLess*			WLC		
	M	*SD*	*n*	*M*	*SD*	*n*	*M*	*SD*	*n*
PSS-10									
Baseline	21.26	7.22	54	21.15	6.09	53	20.23	6.38	48
Post	17.46	6.16	41	19.67	5.66	42	20.06	6.56	47
Follow-up	16.20	5.91	40	17.49	6.03	43	18.58	6.05	45
ERSQ-27									
Baseline	59.8	15.72	54	63.74	16.74	53	62.33	14.76	48
Post	72.85	16.03	41	71.69	14.5	42	63.28	16.65	47
Follow-up	71.7	15.09	40	70.44	17.52	43	63.87	16.07	45
PHQ-9									
Baseline	9.91	4.84	54	9.19	4.99	53	8.75	4.43	48
Post	8	4.2	41	8.29	5.64	42	8.49	3.93	47
Follow-up	7.93	4.93	40	7	4.91	43	7.91	3.52	45
WHO-5									
Baseline	10.39	5.18	54	10.94	3.99	53	11.33	4.37	48
Post	12.1	4.75	41	12.14	3.98	42	11.04	4.09	47
Follow-up	12.65	5.31	40	12.93	4.45	43	11.27	4.36	45

Note. *MT-StressLess* + biofeedback = Mentalis StressLess app-based intervention condition with heart rate-based biofeedback; *MT-StressLess* = Mentalis StressLess app-based intervention condition without heart rate-based biofeedback; WLC = waitlist control; PSS-10 = Perceived Stress Scale; ERSQ-27 = Emotion Regulation Skills Questionnaire; PHQ-9 = Patient Health Questionnaire; WHO-5 = WHO-Five Well-Being Index.

Table S11

Results of the Linear Mixed Model for Perceived Stress Scale (PSS-10) with Fixed and Random Effects: Comparison of MT-StressLess and MT-StressLess + Biofeedback Against Waitlist Control Condition

Variables	Estimates	SE	95% CI Lower	95% CI Upper	t-value	*p*
Intercept	20.23	0.92	18.42	22.03	21.96	< 0.001
MT-StressLess + biofeedback	1.03	1.27	-1.45	3.51	0.81	0.417
MT-StressLess	0.92	1.27	-1.57	3.41	0.72	0.47
Postintervention	-0.24	0.75	-1.72	1.24	-0.32	0.751
Follow-up	-1.97	0.77	-3.48	-0.47	-2.58	0.011
MT-StressLess + biofeedback × Postintervention	-3.58	1.1	-5.72	-1.43	-3.27	0.001
MT-StressLess × Post-assessment	-1.53	1.09	-3.66	0.6	-1.41	0.161
MT-StressLess + biofeedback × Follow-up	-3.07	1.11	-5.24	-0.89	-2.77	0.006
MT-StressLess × Follow-up	-1.95	1.09	-4.09	0.2	-1.78	0.076
Intercept Variance (τ00. id)	27.28					
Residual Variance (σ^2)	13.44					
Intraclass Correlation Coefficient (ICC)	0.67					
Number of Clusters (N id)	155					
Number of Observations	413					

Note. *MT-StressLess* + biofeedback = Mentalis StressLess app-based intervention condition with heart rate-based biofeedback; *MT-StressLess* = Mentalis StressLess app-based intervention condition without heart rate-based biofeedback.

Table S12

Results of the Linear Mixed Model for Perceived Stress Scale (PSS-10) with Fixed and Random Effects: Comparison of MT-StressLess and MT-StressLess + Biofeedback

Variables	Estimates	SE	95% CI Lower	95% CI Upper	t-value	*p*
Intercept	21.15	0.88	19.43	22.87	24.13	< 0.001
WLC	-0.92	1.27	-3.42	1.58	-0.72	0.47
MT-StressLess + biofeedback	0.11	1.23	-2.31	2.53	0.09	0.93
Postintervention	-1.77	0.78	-3.3	-0.25	-2.26	0.025
Follow-up	-3.92	0.78	-5.44	-2.4	-5.04	< 0.001
WLC × Postintervention	1.53	1.09	-0.6	3.66	1.41	0.16
MT-StressLess + biofeedback × Postintervention	-2.05	1.12	-4.24	0.14	-1.84	0.068
WLC × Follow-up	1.95	1.09	-0.18	4.08	1.78	0.076
MT-StressLess + biofeedback × Follow-up	-1.12	1.12	-3.3	1.06	-1	0.317
Intercept Variance (τ00. id)	27.28					
Residual Variance (σ^2)	13.44					
Intraclass Correlation Coefficient (ICC)	0.67					
Number of Clusters (N id)	155					
Number of Observations	413					

Note. *MT-StressLess* + biofeedback = Mentalis StressLess app-based intervention condition with heart rate-based biofeedback; *MT-StressLess* = Mentalis StressLess app-based intervention condition without heart rate-based biofeedback; WLC = waitlist control.

Table S13

Per-Protocol Analysis: Results of the Linear Mixed Model for Perceived Stress Scale (PSS-10) with Fixed and Random Effects: Comparison of MT-StressLess and MT-StressLess + Biofeedback Against Waitlist Control Condition

Variables	Estimates	SE	95% CI Lower	95% CI Upper	t-value	*p*
Intercept	20.23	0.90	18.47	21.98	22.59	< 0.001
MT-StressLess + biofeedback	0.97	1.26	-1.49	3.44	0.77	0.440
MT-StressLess	1.15	1.27	-1.34	3.65	0.91	0.366
Postintervention	-0.24	0.75	-1.71	1.23	-0.32	0.752
Follow-up	-1.97	0.76	-3.46	-0.47	-2.58	0.010
MT-StressLess + biofeedback × Postintervention	-3.56	1.09	-5.70	-1.42	-3.26	0.001
MT-StressLess × Post-assessment	-1.61	1.09	-3.74	0.53	-1.48	0.141
MT-StressLess + biofeedback × Follow-up	-3.05	1.11	-5.22	-0.88	-2.76	0.006
MT-StressLess × Follow-up	-2.03	1.09	-4.17	0.11	-1.86	0.065
Intercept Variance (τ00. id)	25.18					
Residual Variance (σ^2)	13.31					
Intraclass Correlation Coefficient (ICC)	0.65					
Number of Clusters (N id)	144					
Number of Observations	402					

Note. *MT-StressLess* + biofeedback = Mentalis StressLess app-based intervention condition with heart rate-based biofeedback; *MT-StressLess* = Mentalis StressLess app-based intervention condition without heart rate-based biofeedback.

Table S14

Per-Protocol Analysis: Results of the Linear Mixed Model for Perceived Stress Scale (PSS-10) with Fixed and Random Effects: Comparison of MT-StressLess and MT-StressLess + Biofeedback

Variables	Estimates	SE	95% CI Lower	95% CI Upper	t-value	*p*
Intercept	21.38	0.91	19.61	23.16	23.63	< 0.001
WLC	-1.15	1.27	-3.65	1.34	-0.91	0.366
MT-StressLess + biofeedback	-0.18	1.27	-2.66	2.30	-0.14	0.888
Postintervention	-1.84	0.79	-3.39	-0.30	-2.34	0.020
Follow-up	-3.99	0.78	-5.52	-2.46	-5.11	< 0.001
WLC × Postintervention	1.61	1.09	-0.53	3.74	1.48	0.141
MT-StressLess + biofeedback × Postintervention	-1.95	1.12	-4.15	0.24	-1.75	0.082
WLC × Follow-up	2.03	1.09	-0.11	4.17	1.86	0.065
MT-StressLess + biofeedback × Follow-up	-1.03	1.12	-3.22	1.17	-0.92	0.360
Intercept Variance (τ00. id)	25.18					
Residual Variance (σ^2)	13.31					
Intraclass Correlation Coefficient (ICC)	0.65					
Number of Clusters (N id)	144					
Number of Observations	402					

Note. *MT-StressLess* + biofeedback = Mentalis StressLess app-based intervention condition with heart rate-based biofeedback; *MT-StressLess* = Mentalis StressLess app-based intervention condition without heart rate-based biofeedback; WLC = waitlist control.

Table S15

Results of the Linear Mixed Model for Perceived Stress Scale (PSS-10) with Fixed and Random Effects: Comparison of MT-StressLess and MT-StressLess + Biofeedback Against Waitlist Control, Controlling for Sex and Age

Variables	Estimates	SE	95% CI Lower	95% CI Upper	t-value	*p*
Intercept	14.61	2.03	10.63	18.60	7.19	< 0.001
MT-StressLess + biofeedback	1.10	1.24	-1.34	3.53	0.88	0.378
MT-StressLess	0.55	1.24	-1.89	2.99	0.44	0.660
Postintervention	-0.26	0.76	-1.74	1.22	-0.35	0.729
Follow-up	-2.00	0.77	-3.51	-0.50	-2.61	0.010
Sex_2 (Female)	3.91	1.14	1.68	6.14	3.44	0.001
Sex_3 (Diverse)	-0.90	5.57	-11.81	10.01	-0.16	0.871
Age	0.11	0.06	0.00	0.23	1.92	0.057
MT-StressLess + biofeedback × Postintervention	-3.58	1.10	-5.73	-1.43	-3.27	0.001
MT-StressLess × Post-assessment	-1.50	1.09	-3.64	0.63	-1.38	0.169
MT-StressLess + biofeedback × Follow-up	-3.05	1.11	-5.22	-0.88	-2.75	0.006
MT-StressLess × Follow-up	-1.91	1.09	-4.06	0.23	-1.75	0.081
Intercept Variance (τ00. id)	25.12					
Residual Variance (σ^2)	13.45					
Intraclass Correlation Coefficient (ICC)	0.65					
Number of Clusters (N id)	155					
Number of Observations	413					

Note. *MT-StressLess* + biofeedback = Mentalis StressLess app-based intervention condition with heart rate-based biofeedback; *MT-StressLess* = Mentalis StressLess app-based intervention condition without heart rate-based biofeedback.

Table S16

Results of the Linear Mixed Model for Perceived Stress Scale (PSS-10) with Fixed and Random Effects: Comparison of MT-StressLess and MT-StressLess + Biofeedback, Controlling for Sex and Age

Variables	Estimates	SE	95% CI Lower	95% CI Upper	t-value	*p*
Intercept	14.61	2.03	10.63	18.60	7.19	< 0.001
WLC	1.10	1.24	-1.34	3.53	0.88	0.378
MT-StressLess	0.55	1.24	-1.89	2.99	0.44	0.660
Postintervention	-0.26	0.76	-1.74	1.22	-0.35	0.729
Follow-up	-2.00	0.77	-3.51	-0.50	-2.61	0.010
Sex_2 (Female)	3.91	1.14	1.68	6.14	3.44	0.001
Sex_3 (Diverse)	-0.90	5.57	-11.81	10.01	-0.16	0.871
Age	0.11	0.06	0.00	0.23	1.92	0.057
WLC × Postintervention	-3.58	1.10	-5.73	-1.43	-3.27	0.001
MT-StressLess + biofeedback × Post-assessment	-1.50	1.09	-3.64	0.63	-1.38	0.169
WLC × Follow-up	-3.05	1.11	-5.22	-0.88	-2.75	0.006
MT-StressLess + biofeedback × Follow-up	-1.91	1.09	-4.06	0.23	-1.75	0.081
Intercept Variance (τ00. id)	25.12					
Residual Variance (σ^2)	13.45					
Intraclass Correlation Coefficient (ICC)	0.65					
Number of Clusters (N id)	155					
Number of Observations	413					

Note. *MT-StressLess* + biofeedback = Mentalis StressLess app-based intervention condition with heart rate-based biofeedback; *MT-StressLess* = Mentalis StressLess app-based intervention condition without heart rate-based biofeedback; WLC = waitlist control.

Table S17

Results of the Linear Mixed Model for Emotion Regulation Skills Questionnaire (ERSQ-27) with Fixed and Random Effects: Comparison of MT-StressLess and MT-StressLess + Biofeedback Against Waitlist Control Condition

Variables	Estimates	SE	95% CI Lower	95% CI Upper	t-value	*p*
Intercept	62.33	2.31	57.79	66.87	27.01	< 0.001
MT-StressLess + biofeedback	-1.4	3.19	-7.66	4.86	-0.8	0.425
MT-StressLess	-2.54	3.17	-8.78	3.71	-0.44	0.66
Postintervention	1.08	1.96	-2.78	4.93	0.55	0.583
Follow-up	1.53	1.99	-2.38	5.44	0.77	0.442
MT-StressLess + biofeedback × Postintervention	7.85	2.82	2.32	13.37	2.78	0.006
MT-StressLess × Post-assessment	11.42	2.84	5.84	17	4.03	< 0.001
MT-StressLess + biofeedback × Follow-up	5.9	2.83	0.34	11.46	2.08	0.031
MT-StressLess × Follow-up	9.67	2.87	4.02	15.32	3.37	< 0.001
Intercept Variance (τ00. id)	165.51					
Residual Variance (σ^2)	90.21					
Intraclass Correlation Coefficient (ICC)	0.65					
Number of Clusters (N id)	155					
Number of Observations	413					

Note. *MT-StressLess* + biofeedback = Mentalis StressLess app-based intervention condition with heart rate-based biofeedback; *MT-StressLess* = Mentalis StressLess app-based intervention condition without heart rate-based biofeedback.

Table S18

Results of the Linear Mixed Model for Emotion Regulation Skills Questionnaire (ERSQ-27) with Fixed and Random Effects: Comparison of MT-StressLess and MT-StressLess + Biofeedback

Variables	Estimates	SE	95% CI Lower	95% CI Upper	t-value	*p*
Intercept	63.74	2.20	59.43	68.04	29.02	< 0.001
WLC	-1.40	3.19	-7.67	4.86	-0.44	0.660
MT-StressLess + biofeedback	-3.94	3.09	-10.00	2.12	-1.27	0.204
Postintervention	8.92	2.03	4.92	12.93	4.39	< 0.001
Follow-up	7.42	2.01	3.48	11.36	3.69	< 0.001
WLC × Postintervention	-7.85	2.82	-13.37	-2.33	-2.78	0.006
MT-StressLess + biofeedback × Postintervention	3.58	2.89	-2.09	9.24	1.24	0.217
WLC × Follow-up	-5.90	2.83	-11.45	-0.34	-2.08	0.038
MT-StressLess + biofeedback × Follow-up	3.77	2.89	-1.91	9.45	1.31	0.193
Intercept Variance (τ00. id)	165.51					
Residual Variance (σ^2)	90.21					
Intraclass Correlation Coefficient (ICC)	0.65					
Number of Clusters (N id)	155					
Number of Observations	413					

Note. *MT-StressLess* + biofeedback = Mentalis StressLess app-based intervention condition with heart rate-based biofeedback; *MT-StressLess* = Mentalis StressLess app-based intervention condition without heart rate-based biofeedback.

Table S19

Results of the Linear Mixed Model for WHO-5 Well-Being Index (WHO-5) with Fixed and Random Effects: Comparison of MT-StressLess and MT-StressLess + Biofeedback Against Waitlist Control Condition

Variables	Estimates	SE	95% CI Lower	95% CI Upper	t-value	*p*
Intercept	11.33	0.65	10.05	12.61	17.36	< 0.001
MT-StressLess + biofeedback	-0.94	0.9	-2.7	0.81	-1.05	0.294
MT-StressLess	-0.39	0.9	-2.16	1.38	-0.43	0.666
Postintervention	-0.3	0.53	-1.33	0.74	-0.56	0.575
Follow-up	0.1	0.53	-0.95	1.15	0.19	0.85
MT-StressLess + biofeedback × Postintervention	2.14	0.76	0.64	3.63	2.8	0.006
MT-StressLess × Post-assessment	1.52	0.76	0.03	3.01	2	0.043
MT-StressLess + biofeedback × Follow-up	2.2	0.77	0.69	3.71	2.84	0.005
MT-StressLess × Follow-up	1.91	0.76	0.41	3.41	2.51	0.012
Intercept Variance (τ00. id)	13.92					
Residual Variance (σ^2)	6.54					
Intraclass Correlation Coefficient (ICC)	0.68					
Number of Clusters (N id)	155					
Number of Observations	413					

Note. *MT-StressLess* + biofeedback = Mentalis StressLess app-based intervention condition with heart rate-based biofeedback; *MT-StressLess* = Mentalis StressLess app-based intervention condition without heart rate-based biofeedback.

Table S20

Results of the Linear Mixed Model for WHO-5 Well-Being Index (WHO-5) with Fixed and Random Effects: Comparison of MT-StressLess and MT-StressLess + Biofeedback

Variables	Estimates	SE	95% CI Lower	95% CI Upper	t-value	*p*
Intercept	10.94	0.62	9.72	12.16	17.61	< 0.001
WLC	0.39	0.9	-1.37	2.16	0.43	0.666
MT-StressLess + biofeedback	-0.55	0.87	-2.27	1.16	-0.63	0.527
Postintervention	1.23	0.55	0.15	2.3	2.24	0.026
Follow-up	2.01	0.54	0.94	3.08	3.7	< 0.001
WLC × Postintervention	-1.52	0.76	-2.98	-0.07	-2	0.046
MT-StressLess + biofeedback × Postintervention	0.61	0.78	-0.92	2.15	0.79	0.431
WLC × Follow-up	-1.91	0.76	-3.41	-0.41	-2.51	0.013
MT-StressLess + biofeedback × Follow-up	0.29	0.78	-1.26	1.84	0.37	0.713
Intercept Variance (τ00. id)	13.92					
Residual Variance (σ^2)	6.54					
Intraclass Correlation Coefficient (ICC)	0.68					
Number of Clusters (N id)	155					
Number of Observations	413					

Note. *MT-StressLess* + biofeedback = Mentalis StressLess app-based intervention condition with heart rate-based biofeedback; *MT-StressLess* = Mentalis StressLess app-based intervention condition without heart rate-based biofeedback; WLC = waitlist control.

References

1 Klein, E. M. *et al.* The German version of the Perceived Stress Scale–psychometric characteristics in a representative German community sample. *BMC Psychiatry* **16**, 159 (2016). https://doi.org/10.1186/s12888-016-0875-9

2 Cohen, S. & Williamson, G. in *The social psychology of health* (eds S Spacapan & S Oskamp) 31–67 (Sage Publications, Inc, 1988).

3 Berking, M. & Znoj, H. Entwicklung und Validierung eines Fragebogens zur standardisierten Selbsteinschätzung emotionaler Kompetenzen (SEK-27). *Zeitschrift für Psychiatrie, Psychologie und Psychotherapie* **56**, 141-153 (2008). https://doi.org/10.1024/1661-4747.56.2.141

4 Berking, M. *et al.* Affect regulation training reduces symptom severity in depression - A randomized controlled trial. *PloS one* **14**, e0220436-e0220436 (2019). https://doi.org/10.1371/journal.pone.0220436

5 Berking, M. *et al.* Deficits in emotion-regulation skills predict alcohol use during and after cognitive–behavioral therapy for alcohol dependence. *Journal of consulting and clinical psychology* **79**, 307 (2011). https://doi.org/10.1037/a0023421

6 Berking, M., Eichler, E., Naumann, E. & Svaldi, J. The efficacy of a transdiagnostic emotion regulation skills training in the treatment of binge-eating disorder—Results from a randomized controlled trial. *British Journal of Clinical Psychology* (2022). https://doi.org/10.1111/bjc.12371

7 Martin, A., Rief, W., Klaiberg, A. & Braehler, E. Validity of the brief patient health questionnaire mood scale (PHQ-9) in the general population. *General Hospital Psychiatry* **28**, 71-77 (2006). https://doi.org/10.1016/j.genhosppsych.2005.07.003

8 Brähler, E., Mühlan, H., Albani, C. & Schmidt, S. Teststatistische prüfung und normierung der deutschen versionen des EUROHIS-QOL lebensqualität-Index und des WHO-5 wohlbefindens-index. *Diagnostica* **53**, 83-96 (2007). https://doi.org/10.1026/0012-1924.53.2.83

9 Rummel, B. *System Usability Scale – jetzt auch auf Deutsch.*, https://community.sap.com/t5/additional-blogs-by-sap/system-usability-scale-jetzt-auch-auf-deutsch/ba-p/13487686 (2016).
